# *PhWRKY23* Positively Contributes to Herbivore Resistance and Is Associated with Phytohormone and Defense-Related Responses in *Populus hopeiensis*

**DOI:** 10.3390/plants15162483

**Published:** 2026-08-16

**Authors:** Qi Zhang, Jiaxin Liu, Yu-e Bai, Linlin Pang, Shaobin Zhang, Dongying Geng, Jia Liu, Aoga Li

**Affiliations:** College of Forestry, Inner Mongolia Agricultural University, Hohhot 010018, China

**Keywords:** *Populus hopeiensis*, *PhWRKY23*, *Spodoptera litura*, herbivore resistance, jasmonic acid, JA-Ile, salicylic acid, yeast two-hybrid

## Abstract

*Populus hopeiensis* is an important native poplar species in northern China, but herbivorous insect damage seriously affects its growth and ecological function. WRKY transcription factors play important roles in plant stress responses; however, the function of WRKY23 homologs in woody plant resistance to chewing herbivores remains unclear. In this study, a herbivore-responsive WRKY transcription factor gene, *PhWRKY23*, was identified from *P. hopeiensis*. *PhWRKY23* expression was significantly induced by *Spodoptera litura* feeding, with a maximum increase of approximately 69.71-fold the control level at the highest damage level, and the encoded protein was predominantly localized in the nucleus. To investigate its function, *PhWRKY23*-overexpressing and RNA interference transgenic lines were generated. In the choice feeding assay, the consumed leaf area of *PhWRKY23*-overexpressing plants was approximately 85.5% lower than that of WT plants after 8 h. In the no-choice feeding assay, the total larval mass after 6 d was approximately 38.8% lower in larvae fed on overexpression plants and 51.0% higher in larvae fed on RNAi plants than in those fed on WT plants. Physiological analysis showed that RNAi plants accumulated significantly more MDA than WT and overexpression plants, whereas overexpression plants had higher chlorophyll a, chlorophyll b, and carotenoid contents than the other genotypes. Phytohormone analysis further showed that *PhWRKY23*-overexpressing plants accumulated higher levels of jasmonic acid, jasmonoyl-L-isoleucine, and salicylic acid, whereas abscisic acid showed no significant difference among genotypes. Yeast two-hybrid screening identified several candidate PhWRKY23-interacting proteins, and pairwise validation confirmed that PhWRKY23 interacted with PhDOX1 in yeast. These results indicate that *PhWRKY23* positively contributes to herbivore resistance in *P. hopeiensis* and that this resistance phenotype is associated with changes in JA, JA-Ile, and SA accumulation and defense-related physiological traits.

## 1. Introduction

Woody plants are continuously exposed to diverse biotic stresses throughout their long life cycle, among which insect herbivory is one of the major constraints affecting growth, productivity, and ecological adaptation. Poplars (*Populus* spp.) are widely distributed forest trees with significant ecological and economic value. They have also emerged as model woody plants for functional genomics because of their relatively small genomes, rapid growth, and well-established genetic transformation systems [1]. Plant responses to herbivorous insects involve coordinated signaling, transcriptional, metabolic, and physiological changes [2,3,4,5]. Chewing herbivores cause extensive tissue damage and activate wound- and herbivore-associated defense responses, leading to the accumulation of defense-related proteins, metabolites, and signaling molecules [3,4,5,6,7,8,9]. *Spodoptera litura* (Fabricius) is a highly polyphagous lepidopteran pest and is considered a serious threat to many crops and woody plants in the Asia-Pacific region [10]. Therefore, identifying key regulatory genes involved in poplar resistance to chewing insects is essential for elucidating woody plant defense mechanisms and improving insect-resistant germplasm.

Recent studies have shown that poplar defense against insect herbivores is associated with complex biochemical and metabolic responses. In *Populus tremula*, sucking and chewing insect herbivores induce distinct changes in chlorophyll content, polyphenols, volatiles, and other defense-related traits [11]. European aspen (*Populus tremula*) also exhibits dynamic phenolic responses following insect infestation or mechanical damage, suggesting that phenylpropanoid-derived compounds contribute to genotype-dependent defense capacity [12]. In addition, plant secondary metabolites, including phenolics and flavonoids, play important roles in resistance against insect pests [13].

WRKY transcription factors constitute one of the largest plant-specific transcription factor families and are characterized by the conserved WRKYGQK motif and a zinc-finger domain [14,15]. WRKY proteins generally regulate the expression of target genes by binding W-box elements in promoter regions and participate in plant growth and development, phytohormone signaling, and responses to biotic and abiotic stresses [16,17,18,19,20]. Genome-wide analyses have identified numerous WRKY genes in *Populus trichocarpa*, many of which respond to biotic and abiotic stresses, indicating that WRKY transcription factors may play important roles in poplar stress adaptation [16,17]. In recent years, WRKY transcription factors have increasingly been recognized as central regulatory hubs in plant immunity, functioning both as positive and negative regulators and often acting through the integration of phytohormone and MAPK signaling and through interactions with other transcription factors [19,20].

Several WRKY transcription factors have been shown to regulate plant resistance to insect herbivores. In *Nicotiana attenuata*, WRKY3 and WRKY6 coordinate herbivore-induced defense responses, and silencing of these genes compromises resistance to herbivory [21]. In rice (*Oryza sativa* L.), *OsWRKY70* regulates the balance between growth and defense and contributes to resistance against chewing herbivores through hormone-associated signaling pathways [22]. More recently, *BpWRKY6* was reported to enhance insect resistance in *Betula platyphylla* by directly regulating genes involved in jasmonic acid biosynthesis and terpenoid biosynthesis, providing a woody-plant example of WRKY-mediated anti-herbivore defense [23].

WRKY23 is a member of the WRKY transcription factor family with reported roles in both development and stress-associated processes. In *Arabidopsis thaliana*, *AtWRKY23* participates in the establishment of feeding sites during plant–nematode interactions [24]. *AtWRKY23* also regulates root development by modulating local flavonol biosynthesis and auxin distribution [25]. In addition, WRKY23 is involved in the transcriptional network that mediates auxin feedback on PIN polarity [26]. In hybrid poplar (*Populus tremula* × *Populus alba*), functional analysis of *PtWRKY23* indicated that it participates in the response to *Melampsora* (*Melampsora medusae* f. sp. *tremuloidae*) infection and affects defense-associated processes, including redox homeostasis and cell-wall metabolism [27]. However, current evidence for WRKY23 function in woody plants has mainly focused on pathogen defense. Whether WRKY23 homologs contribute to resistance against chewing herbivores in woody plants, particularly in *P. hopeiensis*, remains largely unexplored.

Phytohormones are central regulators of plant defense against herbivores. JA and its bioactive conjugate JA-Ile play crucial roles in wound- and chewing herbivore-induced responses by activating defense-related genes and metabolites [28,29,30,31,32,33,34]. SA also contributes to local and systemic defense [35,36], and its interaction with JA may be antagonistic or cooperative depending on the biological context [37,38,39,40]. WRKY transcription factors can participate in this hormonal crosstalk [19,20]. Therefore, examining JA, JA-Ile, SA, and ABA helps assess whether *PhWRKY23*-associated resistance is accompanied by changes in phytohormone accumulation.

Herbivore resistance is also associated with growth and physiological homeostasis. Defense activation may constrain plant growth because of resource allocation between growth and defense [41]. Herbivore-induced oxidative stress can cause ROS accumulation and membrane lipid peroxidation, which can be evaluated using MDA content and antioxidant enzymes such as SOD [42,43,44]. Photosynthetic pigments contribute to photosynthesis and stress adaptation [45,46,47], whereas PPO activity can reduce tissue nutritional quality by oxidizing phenolic compounds [48,49,50]. Together, these traits provide complementary indicators of defense-associated physiological changes.

Lipid-derived oxylipins contribute to plant stress signaling and defense [51,52,53,54,55]. α-Dioxygenases catalyze fatty acid oxygenation, and *α-DOX1* has been associated with oxidative stress, cell-death regulation, and defense against the potato aphid (*Macrosiphum euphorbiae*) and green peach aphid (*Myzus persicae*) in herbaceous plants [51,52,53]. However, the potential relationship between WRKY23 and DOX-mediated defense in woody plants remains unknown.

*Populus hopeiensis* is an indigenous poplar distributed mainly in northern and northwestern China and exhibits strong drought and cold tolerance [56]. Its regional adaptation makes it a valuable genetic resource for stress-resilient forest-tree breeding in northern China. Compared with genomically well-characterized model poplars such as *P. trichocarpa*, however, the molecular basis of insect defense in *P. hopeiensis* remains poorly understood, making it a relevant species for investigating herbivore-resistance mechanisms in regionally adapted woody plants. In this study, we identified a herbivore-responsive WRKY transcription factor gene, *PhWRKY23*, from *P. hopeiensis* leaves subjected to *S. litura* feeding. We characterized its sequence, phylogenetic relationship, herbivore-induced expression pattern, and subcellular localization. To investigate its biological function, *PhWRKY23*-overexpressing and RNAi lines were generated and evaluated using choice and no-choice feeding assays. Growth traits, defense-related physiological parameters, and the accumulation of JA, JA-Ile, SA, and ABA were also examined. Finally, yeast two-hybrid screening and pairwise validation were performed to identify candidate PhWRKY23-interacting proteins. This study supports a positive role for *PhWRKY23* in the resistance of *P. hopeiensis* to *S. litura* and indicates that the observed resistance phenotype is associated with changes in phytohormone accumulation and defense-related physiological traits. Based on yeast two-hybrid assays, PhDOX1 was identified as a candidate PhWRKY23-interacting protein, providing a basis for future investigation of their potential functional relationship.

## 2. Results

### 2.1. Transcriptome-Based Identification and Molecular Characterization of *PhWRKY23*

**Figure 1:**
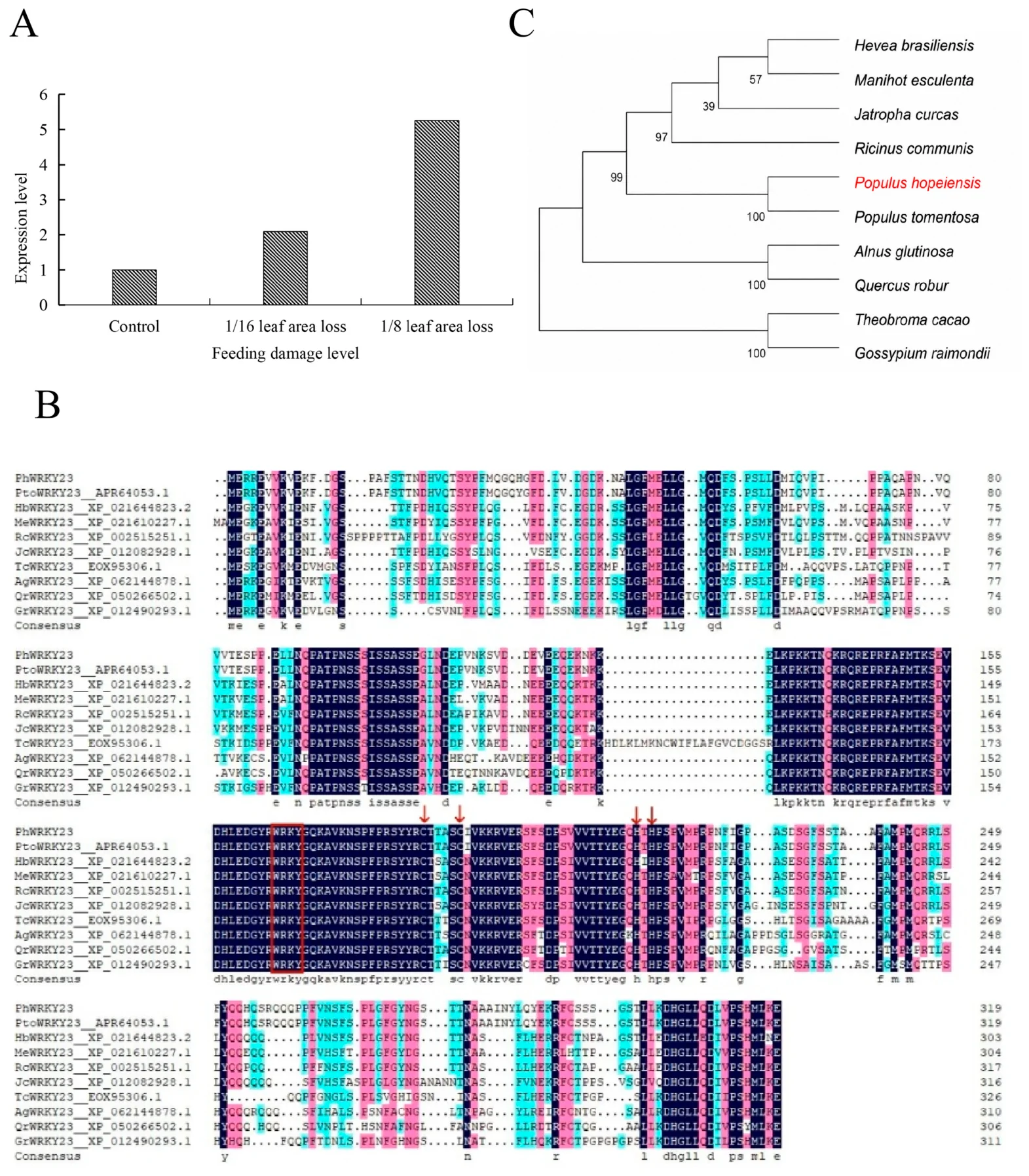
Transcriptome-based identification and molecular characterization of *PhWRKY23.* (**A**) Normalized transcript abundance of *PhWRKY23* in *Populus hopeiensis* leaves under different levels of *Spodoptera litura* feeding damage. The treatments comprised an untreated control and approximately 1/16 and 1/8 leaf-area-loss treatments. (**B**) Multiple sequence alignment of PhWRKY23 and its homologs from different plant species. The characteristic WRKYGQK motif and C_2_H_2_-type zinc-finger motif are indicated by red arrows. (**C**) Phylogenetic analysis of PhWRKY23 and its homologs. The neighbor-joining tree was constructed using 1000 bootstrap replicates. PhWRKY23 is highlighted in red.

Based on our previously generated transcriptomic data from *Populus hopeiensis* leaves subjected to herbivore feeding, an herbivore-responsive WRKY transcription factor gene was identified and designated *PhWRKY23*. The normalized transcript abundance of *PhWRKY23* increased from approximately 1.10 in the untreated control to 2.09 and 5.25 under the 1/16 and 1/8 leaf-area-loss treatments, respectively (Figure 1A). Based on its feeding-responsive expression pattern and the established involvement of WRKY transcription factors in plant defense, *PhWRKY23* was selected for subsequent functional characterization.

The full-length coding sequence of *PhWRKY23* was amplified from *Populus hopeiensis* leaf cDNA. Sequence analysis showed that the coding sequence was 960 bp in length and encoded a protein of 319 amino acids, with a predicted molecular mass of 35.95 kDa and a theoretical isoelectric point of 6.72. InterProScan classified PhWRKY23 as a Group IIc WRKY transcription factor (IPR017396), while Pfam analysis identified a WRKY DNA-binding domain (PF03106) spanning amino acid residues 158–215. This domain contained the characteristic WRKYGQK motif at residues 163–169 and a C_2_H_2_-type zinc-finger motif (C183–X4–C188–X23–H212–X–H214). cNLS Mapper did not identify a high-confidence classical NLS at the default cut-off score of 5.0. However, two low-scoring candidate bipartite NLSs were predicted at residues 135–169 and 162–197, with scores of 4.6 and 4.5, respectively (Figure S1). Multiple sequence alignment further demonstrated that the WRKYGQK and C_2_H_2_-type zinc-finger motifs were conserved among WRKY23 homologs from different plant species (Figure 1B). These sequence characteristics support the classification of PhWRKY23 as a Group IIc WRKY transcription factor.

Phylogenetic analysis showed that PhWRKY23 clustered most closely with the WRKY23 homolog from *Populus tomentosa*, with a bootstrap support value of 100 (Figure 1C). The two *Populus* WRKY23 homologs formed a distinct clade and were further grouped with homologs from *Ricinus communis*, *Jatropha curcas*, *Manihot esculenta*, and *Hevea brasiliensis*. Together, the conserved sequence motifs and phylogenetic relationships supported the classification of PhWRKY23 as a Group IIc WRKY transcription factor.

### 2.2. *PhWRKY23* Expression Under Different Levels of S. litura Feeding Damage and Its Subcellular Localization

**Figure 2:**
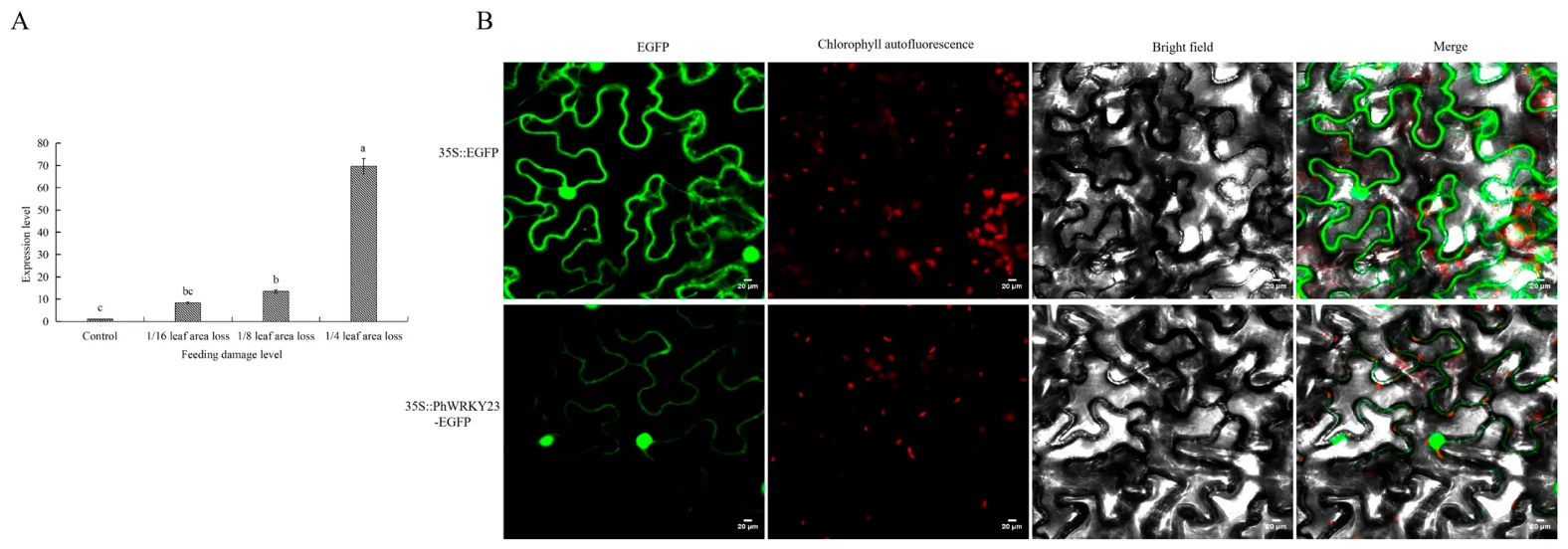
Herbivore-induced expression and subcellular localization of PhWRKY23. (**A**) Relative expression levels of *PhWRKY23* in *Populus hopeiensis* leaves under different levels of *Spodoptera litura* feeding damage, as determined by qRT-PCR. Values are means ± SE of 3 biological replicates. Different lowercase letters indicate significant differences among treatments at *p* < 0.05. (**B**) Subcellular localization of PhWRKY23 in *Nicotiana benthamiana* leaf epidermal cells. Free EGFP was broadly distributed throughout the cells, whereas the PhWRKY23–EGFP fusion protein was predominantly localized in the nucleus, with relatively weak fluorescence also observed in the cytoplasm.

To validate the response of *PhWRKY23* to different levels of herbivore damage, its transcript abundance was examined by qRT-PCR in *Populus hopeiensis* leaves subjected to approximately 1/16, 1/8, and 1/4 leaf-area loss caused by *S. litura* feeding. Compared with the untreated control, *PhWRKY23* transcript abundance increased by approximately 8.28-, 13.45-, and 69.71-fold under the 1/16, 1/8, and 1/4 leaf-area-loss treatments, respectively (Figure 2A). The increase under the 1/16 treatment was not statistically significant, whereas significant increases were observed under the 1/8 and 1/4 treatments. The highest transcript abundance was detected under the 1/4 leaf-area-loss treatment and was significantly higher than that under the other treatments. These results indicate that *PhWRKY23* expression was responsive to *S. litura* feeding and was associated with the severity of feeding damage under the conditions examined. Because the experiment compared different levels of leaf damage rather than sequential sampling time points, these results do not establish the temporal induction pattern of *PhWRKY23*.

To determine the subcellular localization of PhWRKY23, the PhWRKY23–EGFP fusion protein and free EGFP control were transiently expressed in *Nicotiana benthamiana* leaf epidermal cells. The free EGFP signal was broadly distributed throughout the cells, whereas the PhWRKY23–EGFP fluorescence signal was detected predominantly in the nucleus, with relatively weak fluorescence also observed in the cytoplasm (Figure 2B). The weak cytoplasmic fluorescence may represent either background signal or a minor cytoplasmic distribution of the fusion protein. Although cNLS Mapper did not identify a high-confidence classical nuclear localization signal, the observed fluorescence pattern indicated that PhWRKY23 was predominantly, rather than exclusively, localized in the nucleus, consistent with its proposed function as a WRKY transcription factor.

### 2.3. Generation and Molecular Verification of *PhWRKY23* Transgenic P. hopeiensis

**Figure 3:**
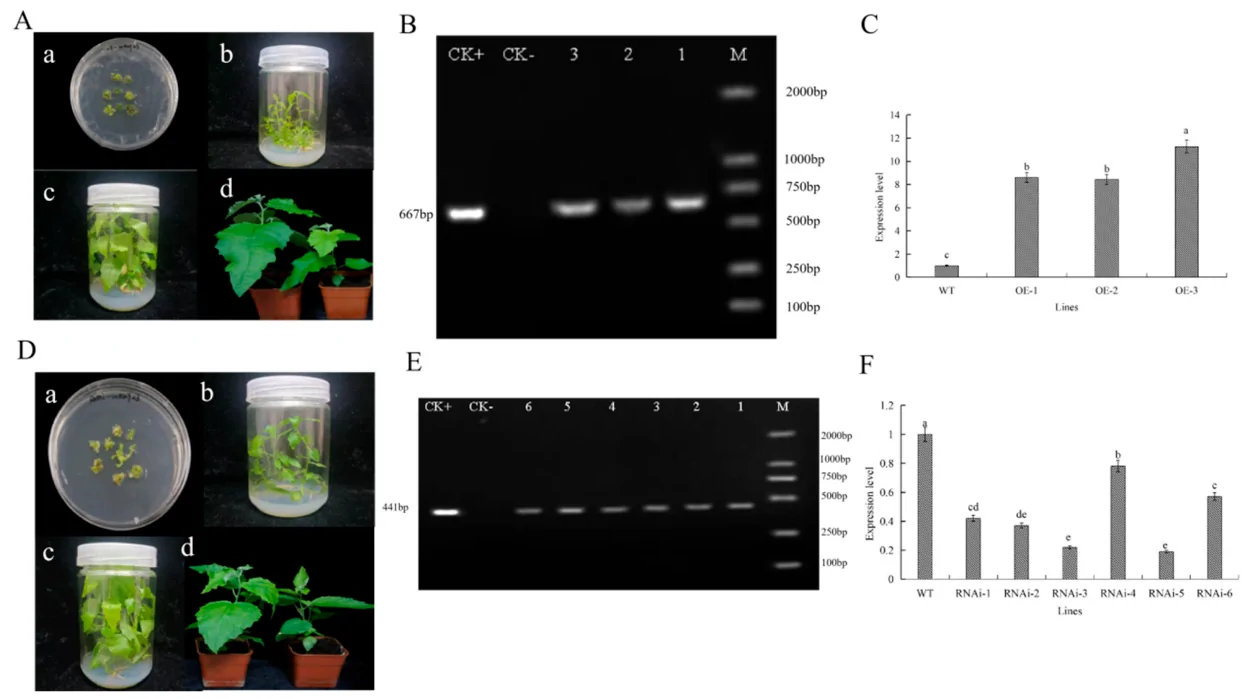
Generation and molecular characterization of *PhWRKY23* transgenic *Populus hopeiensis* lines. (**Aa**–**Ad**) Representative regeneration and growth process of *PhWRKY23*-overexpressing plants, including resistant shoot induction, shoot development, plantlet regeneration, and acclimatization. (**B**) PCR identification of three independent *PhWRKY23*-overexpressing lines. The expected amplification product was 667 bp. CK+, positive control; CK-, negative control; lanes 1–3, independent overexpression lines; M, DNA marker. (**C**) Relative expression levels of *PhWRKY23* in WT and three independent overexpression lines, as determined by qRT-PCR. (**Da**–**Dd**) Representative regeneration and growth process of *PhWRKY23* RNAi plants, including resistant shoot induction, shoot development, plantlet regeneration, and acclimatization. (**E**) PCR identification of six independent *PhWRKY23* RNAi lines. The expected amplification product was 441 bp. CK+, positive control; CK-, negative control; lanes 1–6, independent RNAi lines; M, DNA marker. (**F**) Relative expression levels of *PhWRKY23* in WT and six independent RNAi lines, as determined by qRT-PCR. Data in (**C**,**F**) are presented as means ± SE of three biological replicates (*n* = 3 independently propagated plants per transgenic line or WT group). Each biological replicate was analyzed using three technical replicates. Different lowercase letters indicate significant differences among WT and the individual transgenic lines at *p* < 0.05.

To investigate the biological function of *PhWRKY23*, overexpression and RNA interference constructs were introduced into *P. hopeiensis* through *Agrobacterium*-mediated transformation. Following antibiotic selection and regeneration, resistant shoots were obtained and subsequently cultured for shoot development and rooting. Regenerated plantlets were subsequently acclimatized and transferred to soil. Representative regeneration and growth of the *PhWRKY23*-overexpressing and RNAi plants are shown in Figure 3A and Figure 3D, respectively.

Genomic PCR was performed to verify the presence of the corresponding transgenic elements. The expected 667 bp fragment was detected in 3 independent transgenic overexpression lines (OE-1, OE-2, and OE-3), whereas no corresponding amplification product was detected in the negative control (Figure 3B). Similarly, the expected 441 bp fragment was detected in 6 independent transgenic RNAi lines (RNAi-1–RNAi-6), but not in the negative control (Figure 3E). These results confirmed the successful integration of the corresponding transgenic elements in the regenerated plants.

The transcript abundance of *PhWRKY23* was subsequently examined by qRT-PCR. Compared with WT plants, *PhWRKY23* transcript abundance was significantly increased in all 3 OE lines, reaching approximately 8.43-, 8.61-, and 11.26-fold the WT level in OE-1, OE-2, and OE-3, respectively (Figure 3C). By contrast, *PhWRKY23* transcript abundance was significantly reduced in all six RNAi lines, with relative transcript levels ranging from approximately 0.19 to 0.78 times that of the WT level (Figure 3F). Among these lines, RNAi-3 and RNAi-5 exhibited the strongest suppression of *PhWRKY23* expression. Collectively, these results demonstrate that OE and RNAi lines exhibited consistent directional changes in *PhWRKY23* transcript abundance.

Based on PCR confirmation, reproducible changes in *PhWRKY23* transcript abundance, normal vegetative propagation, and the availability of sufficient clonally propagated plants, OE-1, OE-2, OE-3, RNAi-2, RNAi-3, and RNAi-5 were selected for subsequent growth, physiological, biochemical, phytohormone, and insect-feeding analyses. Multiple independent transgenic lines were included for each construct to evaluate whether the observed functional responses were reproducible across different transformation events. Data from the selected transgenic lines were analyzed separately for the qRT-PCR and growth-trait analyses. For the feeding, physiological, biochemical, and phytohormone analyses, the three selected independent lines were treated as biological replicates of the corresponding OE or RNAi group, as described in Section 4.8, Section 4.9, Section 4.10, Section 4.11 and Section 4.12.

### 2.4. *PhWRKY23* Reduces Leaf Damage and Larval Weight Gain Under S. litura Feeding

**Figure 4:**
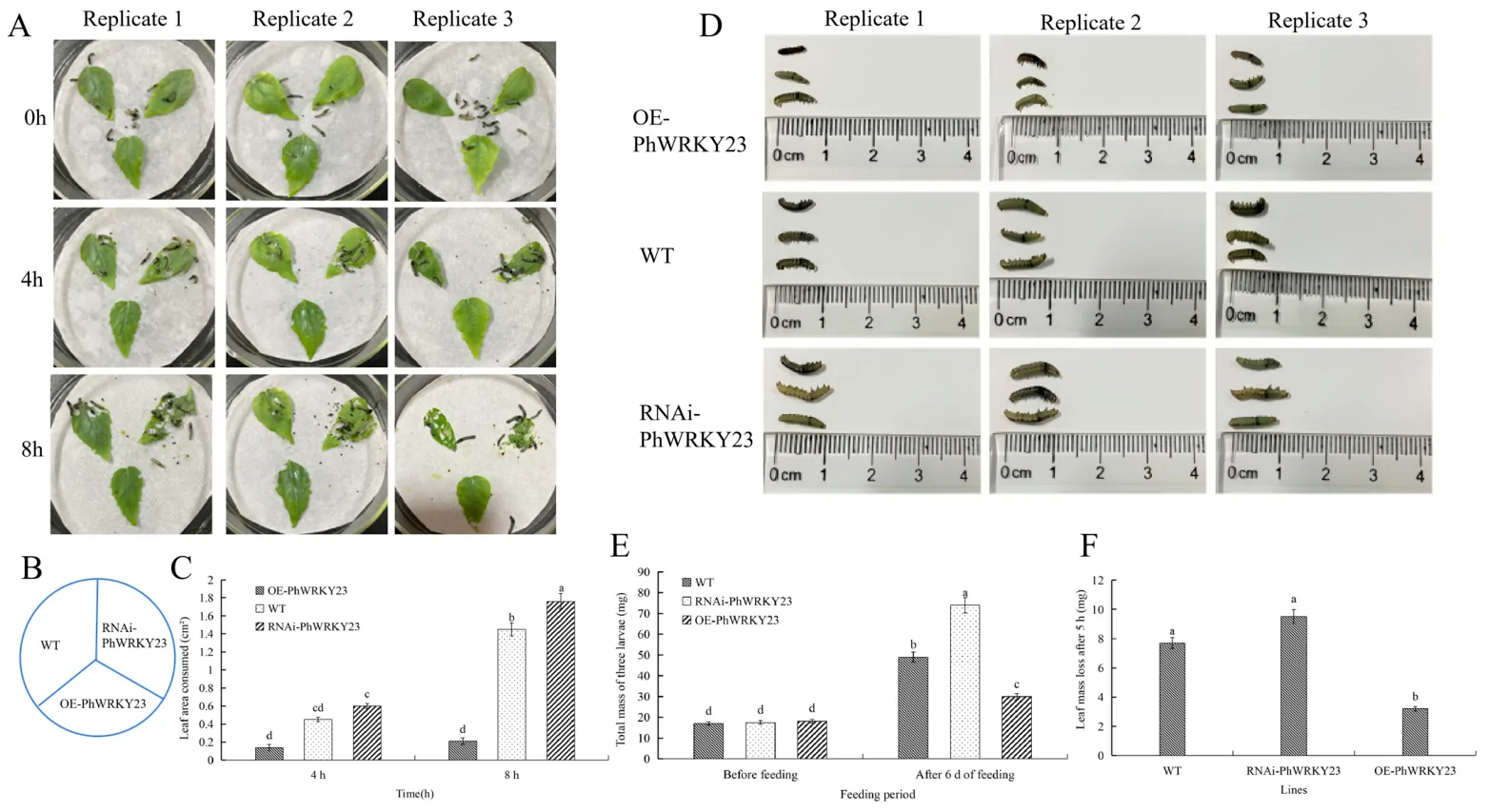
Effects of *PhWRKY23* expression on leaf damage and larval weight gain under *Spodoptera litura* feeding. (**A**) Representative images of the choice feeding assay at 0, 4, and 8 h after larval release. 3 independent biological replicates are shown. (**B**) Schematic diagram showing the arrangement of WT, *PhWRKY23*-overexpressing, and RNAi leaves in the choice feeding assay. (**C**) Quantification of leaf area consumed after 4 and 8 h of choice feeding. (**D**) Representative morphology of *S. litura* larvae after 6 d of feeding on WT, *PhWRKY23*-overexpressing, and RNAi leaves under no-choice conditions. 3 independent biological replicates are shown. (**E**) Total mass of 3 larvae before feeding and after 6 d of feeding on leaves of the indicated genotypes. (**F**) Leaf mass loss after 5 h of feeding. Values are means ± SE of 3 biological replicates. Different lowercase letters indicate significant differences among treatments at *p* < 0.05.

To evaluate the role of *PhWRKY23* in resistance to *S. litura*, choice- and no-choice feeding assays were performed using leaves from WT, *PhWRKY23*-overexpressing, and RNAi plants. Under choice-feeding conditions, leaf damage increased with feeding time, but clear differences were observed among the 3 genotypes (Figure 4A,B). Leaves from the overexpression plants consistently showed the least damage, whereas RNAi leaves were consumed more extensively, with WT leaves displaying an intermediate level of damage.

Quantification of the consumed leaf area further confirmed these differences (Figure 4C). After 4 h of feeding, the consumed leaf area was approximately 0.14 cm^2^ in the overexpression group, compared with approximately 0.45 cm^2^ in the WT group and 0.60 cm^2^ in the RNAi group. After 8 h, the consumed leaf area increased to approximately 0.21, 1.45, and 1.76 cm^2^ in the overexpression, WT, and RNAi groups, respectively. These results indicate that increased *PhWRKY23* expression reduced leaf damage under the choice-feeding conditions examined, whereas suppression of *PhWRKY23* was associated with increased leaf damage.

The effect of *PhWRKY23* expression on larval weight gain was further evaluated using a no-choice feeding assay. After 6 d of feeding, larvae maintained on leaves from the overexpression plants appeared smaller than those maintained on WT leaves, whereas larvae maintained on RNAi leaves appeared largest (Figure 4D). The initial total mass of 3 larvae was similar among the treatments, at approximately 17–18 mg. After 6 d of feeding, the total larval mass differed significantly among treatments, reaching approximately 74 mg in the RNAi group, 49 mg in the WT group, and 30 mg in the overexpression group (Figure 4E). Thus, larvae maintained on leaves from the overexpression plants showed lower weight gain than those maintained on WT leaves, whereas larvae maintained on RNAi leaves showed greater weight gain under the experimental conditions.

Short-term feeding analysis showed a similar pattern. After 5 h of feeding, leaf mass loss was approximately 7.7 mg in WT plants and 9.5 mg in RNAi plants, with no significant difference between these two groups, whereas the overexpression plants showed significantly lower leaf mass loss of approximately 3.2 mg (Figure 4F). Taken together, the observed differences in leaf damage, leaf mass loss, and larval weight gain indicate that *PhWRKY23* positively contributes to the resistance of *P. hopeiensis* against *S. litura* under the experimental conditions examined.

### 2.5. Effects of *PhWRKY23* on Measured Growth Traits and Defense-Related Physiological Responses

**Figure 5:**
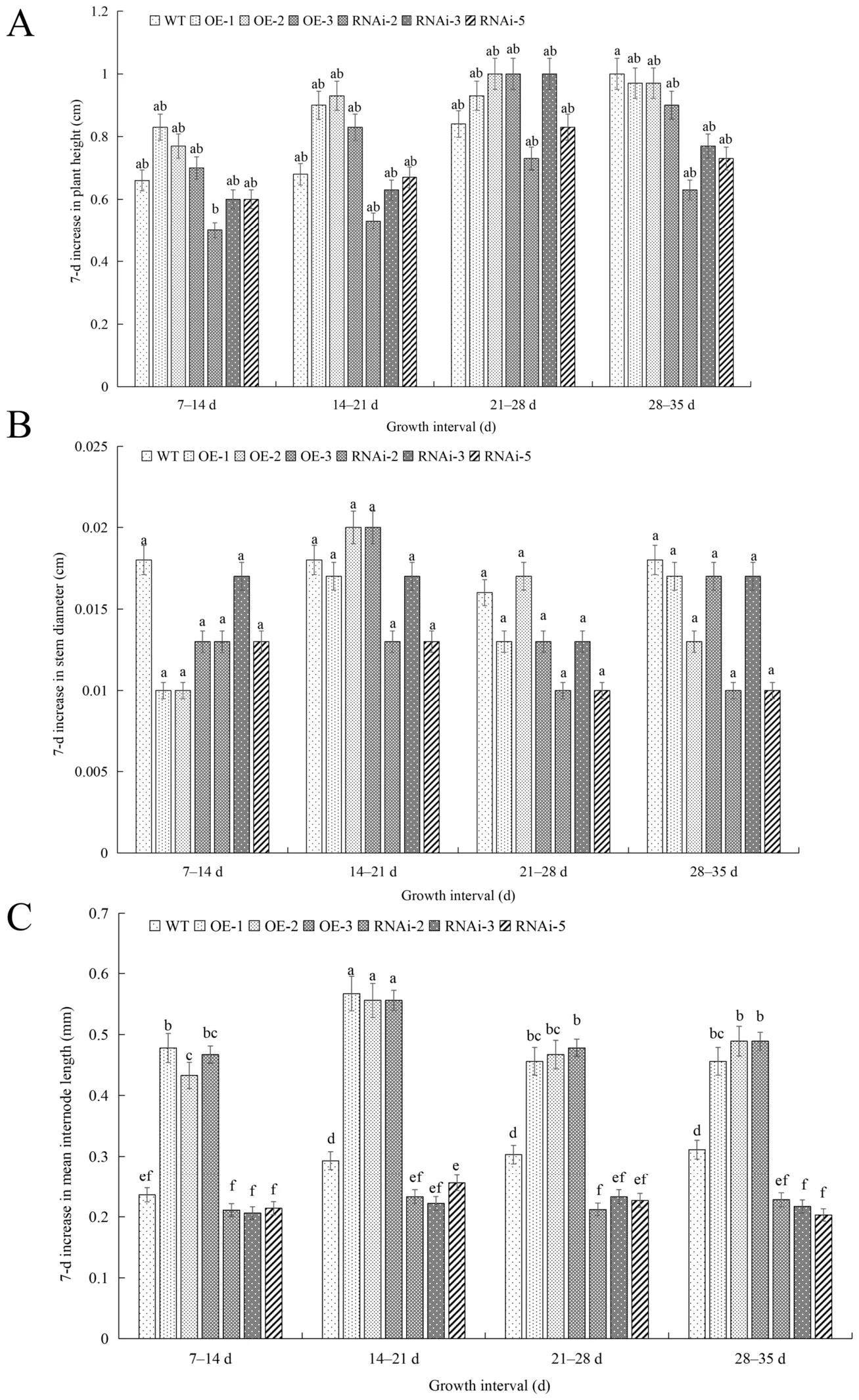
Effects of *PhWRKY23* expression on growth-related traits in *Populus hopeiensis*. (**A**) Seven-day increment in plant height. (**B**) Seven-day increment in basal stem diameter. (**C**) Seven-day increment in mean internode length. Data are presented as means ± SE of 3 biological replicates (*n* = 3 individual plants for each transgenic line and the WT group). Statistical comparisons were performed separately among WT and the individual transgenic lines within each 7-day growth interval. When variances were homogeneous, one-way ANOVA followed by Tukey’s HSD test was used; when variances were unequal, Welch’s ANOVA followed by an appropriate multiple-comparison procedure was used where applicable. Different lowercase letters indicate significant differences among genotypes within the same growth interval at *p* < 0.05.

To evaluate whether altered *PhWRKY23* expression affected the growth traits examined in this study, 7-day increments in plant height, basal stem diameter, and mean internode length were compared among WT, *PhWRKY23*-overexpressing, and RNAi lines over four successive growth intervals. Plant-height increments varied numerically among growth intervals and lines; however, no significant differences were detected between the WT and the individual transgenic lines within any 7-day growth interval (Figure 5A).

Basal stem diameter increments remained relatively stable among WT, overexpression, and RNAi lines throughout the observation period, with no significant differences detected among the examined lines (Figure 5B). Thus, altered *PhWRKY23* expression was not associated with detectable changes in radial stem growth under the conditions and during the observation period examined.

Mean internode-length increments differed among individual lines during some growth intervals (Figure 5C). The three *PhWRKY23*-overexpressing lines generally showed greater internode-length increments, particularly during the 14–21-day interval, whereas the RNAi lines generally maintained relatively low increments and WT plants showed intermediate values. These results indicate that altered *PhWRKY23* expression was associated with differences in internode-length increments, but not with detectable differences in plant-height or basal-stem-diameter increments.

Because the present evaluation was limited to plant height, basal stem diameter, and mean internode length over the examined period, it does not provide a comprehensive assessment of plant growth or fitness. Nevertheless, under the experimental conditions and based on the growth parameters evaluated in this study, *PhWRKY23* overexpression did not cause an obvious growth penalty.

**Figure 6:**
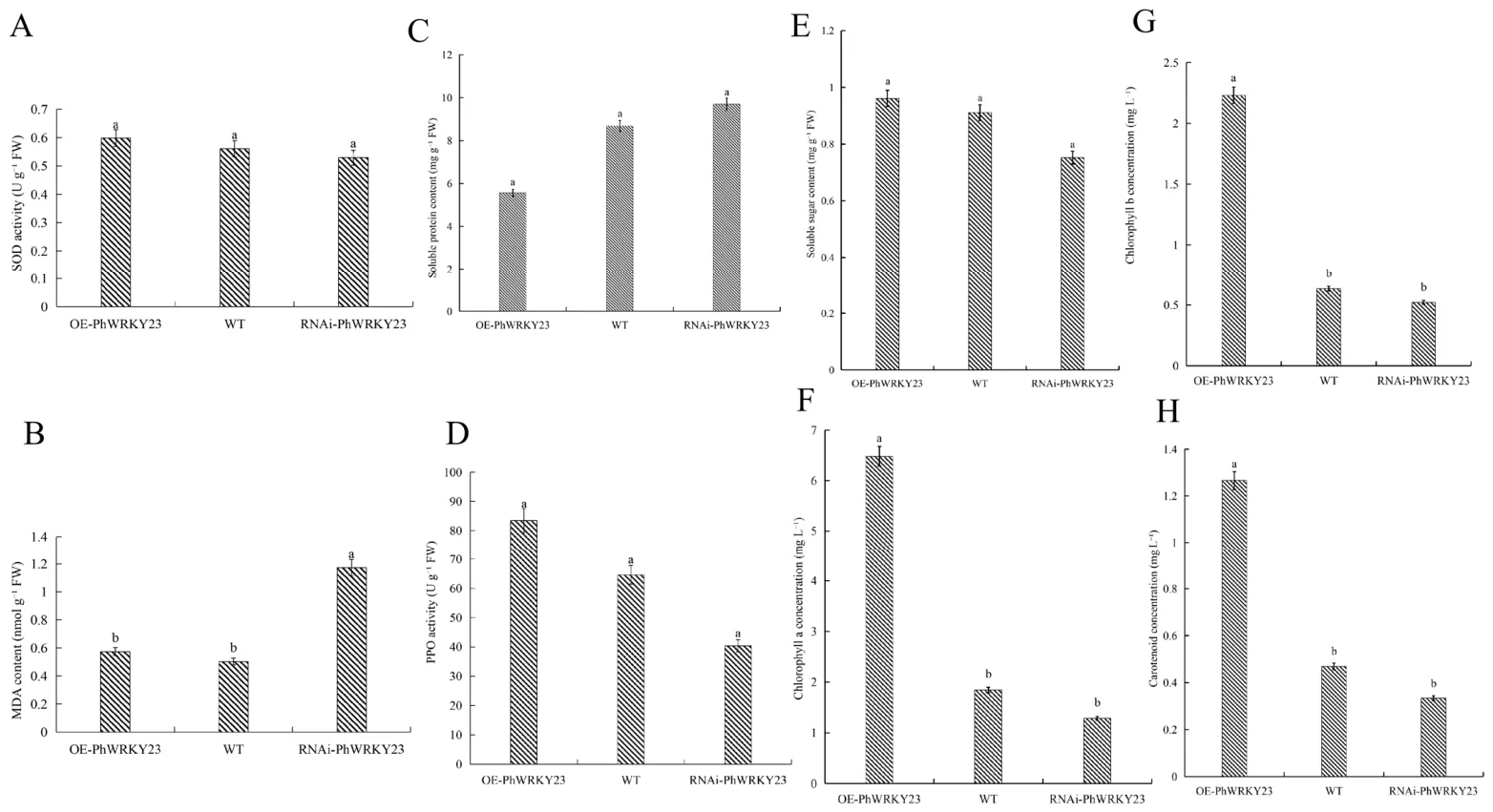
Physiological and biochemical traits of WT, *PhWRKY23*-overexpressing, and RNAi *Populus hopeiensis* plants. (**A**) Superoxide dismutase (SOD) activity. (**B**) Malondialdehyde (MDA) content. (**C**) Soluble protein content. (**D**) Polyphenol oxidase (PPO) activity. (**E**) Soluble sugar content. (**F**) Chlorophyll a content. (**G**) Chlorophyll b content. (**H**) Carotenoid content. Data are presented as means ± SE. Different lowercase letters indicate significant differences among groups at *p* < 0.05. The overexpression and RNAi groups each comprised three selected independent transgenic lines, whereas the WT group comprised three independently grown plants as biological replicates.

To examine whether altered *PhWRKY23* expression was associated with differences in the measured physiological and biochemical traits, SOD activity, MDA content, soluble protein content, PPO activity, soluble sugar content, and photosynthetic pigment contents were compared among WT, *PhWRKY23*-overexpressing, and RNAi plants. SOD activity did not differ significantly among the three groups (Figure 6A). MDA content was significantly higher in the RNAi plants than in the WT and overexpression plants, whereas no significant difference was detected between WT and the overexpression group (Figure 6B). Soluble protein content and PPO activity also showed no significant differences among the three groups (Figure 6C,D).

Soluble sugar content did not differ significantly among the three groups (Figure 6E). In contrast, significant differences were observed in photosynthetic pigment contents. Chlorophyll a, chlorophyll b, and carotenoid contents were significantly higher in the *PhWRKY23*-overexpressing plants than in WT and RNAi plants, whereas no significant differences were detected between WT and RNAi plants (Figure 6F–H).

Collectively, altered *PhWRKY23* expression was associated with differences in MDA and photosynthetic pigment contents under the conditions examined. RNAi plants had higher MDA content, whereas overexpression plants had higher chlorophyll a, chlorophyll b, and carotenoid contents. By contrast, SOD activity, PPO activity, soluble protein content, and soluble sugar content did not differ significantly among the examined groups. These results describe differences in the measured physiological traits but do not establish that *PhWRKY23* directly regulates oxidative-stress responses or maintains overall physiological homeostasis.

### 2.6. Phytohormone Levels in *PhWRKY23* Transgenic Plants

To examine phytohormone changes in the transgenic plants, the levels of jasmonic acid (JA), jasmonoyl-L-isoleucine (JA-Ile), salicylic acid (SA), and abscisic acid (ABA) were measured in WT, *PhWRKY23*-overexpressing, and RNAi plants.

**Figure 7:**
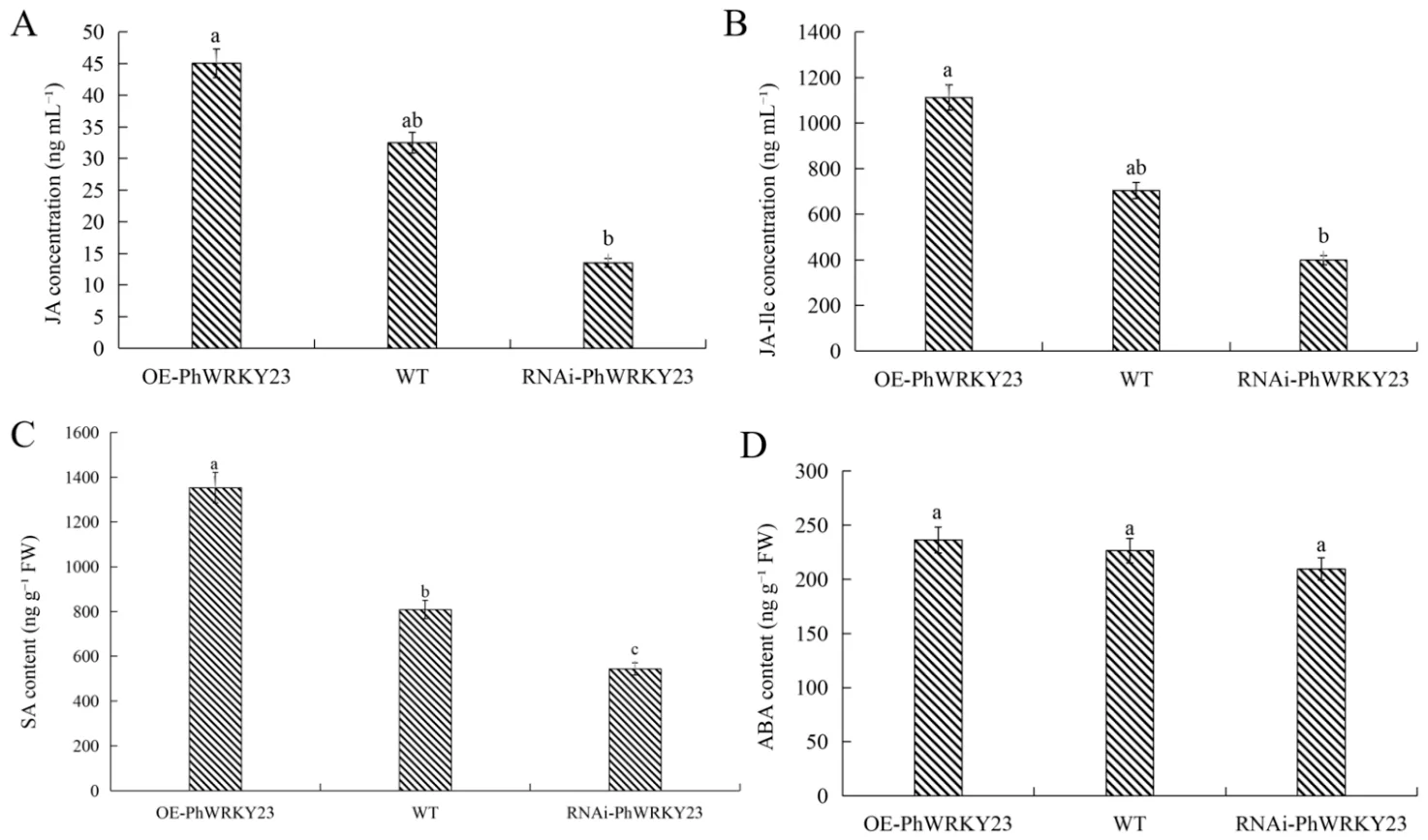
Phytohormone levels in WT, *PhWRKY23*-overexpressing, and RNAi plants of *Populus hopeiensis*. (**A**) Jasmonic acid (JA). (**B**) Jasmonoyl-L-isoleucine (JA-Ile). (**C**) Salicylic acid (SA). (**D**) Abscisic acid (ABA). Data are presented as means ± SE (*n* = 3 biological replicates per group). For the overexpression and RNAi groups, the three selected independent transgenic lines were treated as biological replicates, whereas the WT biological replicates consisted of three independently grown plants. Different lowercase letters indicate significant differences among groups at *p* < 0.05.

JA content was 45.07 ± 3.53 ng mL^−1^ in the overexpression plants, 32.50 ± 0.76 ng mL^−1^ in the WT plants, and 13.52 ± 0.39 ng mL^−1^ in the RNAi plants (Figure 7A). JA content was significantly higher in the overexpression plants than in the RNAi plants, whereas WT plants exhibited an intermediate value and did not differ significantly from either transgenic group.

A similar pattern was observed for JA-Ile (Figure 7B). JA-Ile content was 1112.63 ± 118.91 ng mL^−1^ in the overexpression plants, 705.47 ± 16.34 ng mL^−1^ in WT plants, and 399.38 ± 58.65 ng mL^−1^ in the RNAi plants. The overexpression and RNAi plants differed significantly, whereas WT plants did not differ significantly from either group.

SA content was highest in the overexpression plants (1353.30 ng g^−1^ FW), followed by WT plants (808.84 ng g^−1^ FW) and RNAi plants (543.35 ng g^−1^ FW) (Figure 7C). Thus, SA content exhibited the same overall pattern as JA and JA-Ile.

In contrast, ABA content was relatively similar among the 3 groups, with values of 236.39, 226.59, and 209.61 ng g^−1^ FW in the overexpression, WT, and RNAi plants, respectively (Figure 7D). No significant differences in ABA content were detected among the 3 groups.

Collectively, *PhWRKY23* overexpression was associated with higher levels of JA, JA-Ile, and SA, whereas reduced *PhWRKY23* expression was associated with lower levels of these phytohormones. ABA content did not differ significantly among the examined groups. These findings show an association between *PhWRKY23* expression and phytohormone levels but do not demonstrate direct regulation or causality.

### 2.7. Yeast Two-Hybrid Screening of Candidate *PhWRKY23-Interacting* Proteins

Before library screening, pGBKT7-PhWRKY23 was tested for autoactivation. Weak autoactivation was observed but was effectively suppressed by 5 mM 3-amino-1,2,4-triazole (3-AT) (Figures S2 and S3). Therefore, selective medium supplemented with 5 mM 3-AT was used for subsequent library screening.

Using PhWRKY23 as bait, a yeast two-hybrid screen was performed against a cDNA library prepared from *S. litura*-fed *Populus hopeiensis* leaves. Colonies that grew and turned blue on SD/-Trp/-Leu/-His/-Ade/X-α-gal medium containing 5 mM 3-AT were selected and sequenced. A total of 40 candidate PhWRKY23-interacting proteins were identified (Table S1).

Functional annotation indicated that these candidates were associated with several biological processes, including stress responses, growth and development, protein degradation, photosynthesis, and transport. PhDOX1 was selected for pairwise yeast two-hybrid analysis because it was annotated as a dioxygenase potentially associated with plant stress responses. This selection was based on its functional annotation, and its candidate interaction with PhWRKY23 required further validation.

### 2.8. Pairwise Yeast Two-Hybrid Analysis of *PhWRKY23* and *PhDOX1*

**Figure 8:**
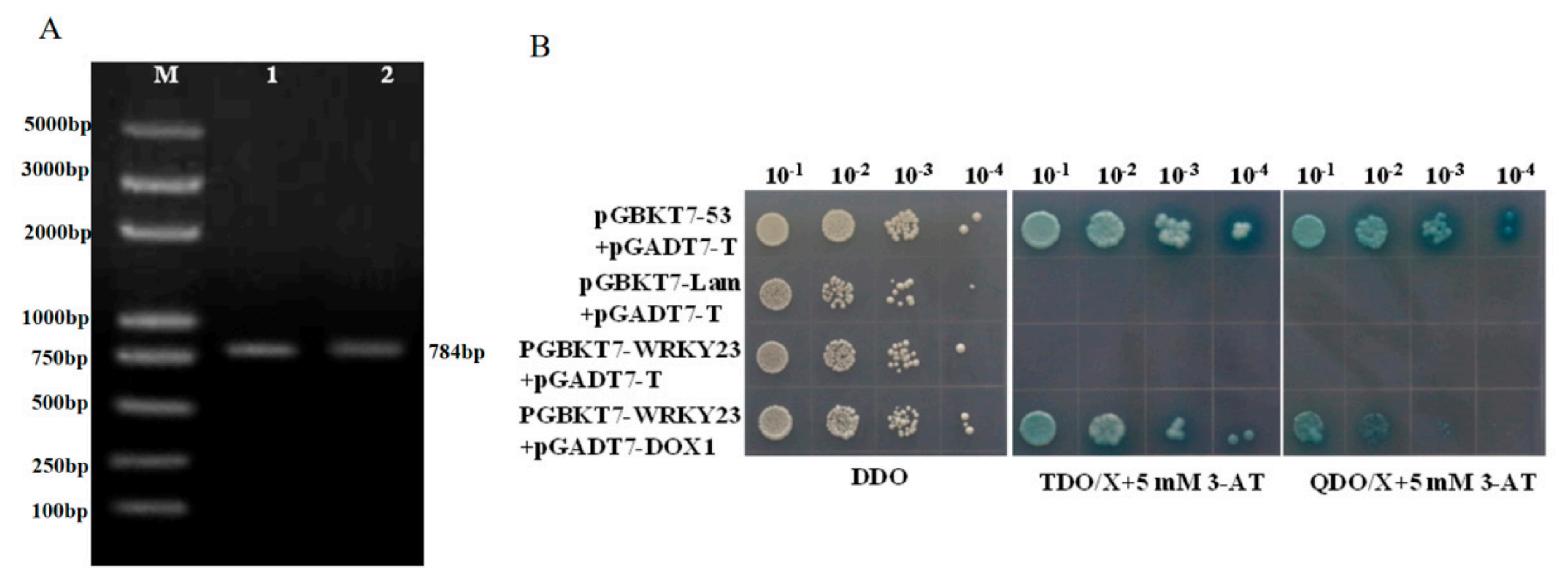
Pairwise yeast two-hybrid analysis of PhWRKY23 and PhDOX1. (**A**) Colony PCR verification of the pGADT7-PhDOX1 construct. M, DNA marker; lanes 1–2, independent positive colonies. The expected amplification product was 784 bp. (**B**) Growth and reporter-gene activation of yeast cells co-transformed with pGBKT7-PhWRKY23 and pGADT7-PhDOX1. The pGBKT7-53/pGADT7-T combination was used as the positive control, pGBKT7-Lam/pGADT7-T as the negative control, and pGBKT7-PhWRKY23/pGADT7-T as the autoactivation control. Yeast cells were tested on DDO, TDO/X, and QDO/X media, with the latter two supplemented with 5 mM 3-AT. DDO, SD/−Trp/−Leu; TDO/X, SD/−Trp/−Leu/−His supplemented with X-α-gal; QDO/X, SD/−Trp/−Leu/−His/−Ade supplemented with X-α-gal.

The coding sequence of *PhDOX1* was amplified from *Populus hopeiensis* leaf cDNA and inserted into the pGADT7 vector. Colony PCR confirmed the presence of the expected insert in the pGADT7-PhDOX1 construct (Figure 8A).

Because the weak autoactivation of pGBKT7-PhWRKY23 was suppressed by 5 mM 3-AT, selective media supplemented with 5 mM 3-AT were used for the pairwise yeast two-hybrid analysis. Yeast cells co-transformed with pGBKT7-PhWRKY23 and pGADT7-PhDOX1 grew on DDO medium and also grew and turned blue on TDO/X and QDO/X media containing 5 mM 3-AT (Figure 8B). The positive control, pGBKT7-53/pGADT7-T, grew and turned blue on the selective media, whereas the negative control, pGBKT7-Lam/pGADT7-T, did not grow. The pGBKT7-PhWRKY23/pGADT7-T combination was included as the autoactivation control. These results indicate that PhWRKY23 interacted with PhDOX1 in the yeast two-hybrid system.

## 3. Discussion

### 3.1. *PhWRKY23* Positively Contributes to Herbivore Resistance in Populus hopeiensis

WRKY transcription factors are key regulatory proteins that connect external stress signals with downstream transcriptional responses and are widely involved in plant immunity, hormone signaling, growth, development, and stress adaptation [16,17,18,19,20]. In the present study, *PhWRKY23* was identified as a herbivore-responsive Group IIc WRKY transcription factor in *Populus hopeiensis*. Its transcript level showed a damage-intensity-associated increase following *S. litura* feeding, with significant induction observed at the higher damage levels, and the PhWRKY23-EGFP fusion protein was predominantly localized in the nucleus. These findings are consistent with the typical characteristics of WRKY transcription factors, which commonly function as nuclear-localized transcriptional regulators in stress-responsive gene networks [16,17,18,19,20].

Functional analyses using transgenic plants provided further evidence that *PhWRKY23* positively contributes to resistance against *S. litura*. In the choice feeding assay, *PhWRKY23*-overexpressing plants showed less leaf consumption, whereas RNAi plants were consumed more extensively. In the no-choice feeding assay, larvae feeding on *PhWRKY23*-overexpressing plants accumulated less mass, whereas those feeding on RNAi plants accumulated more mass. These results indicate that *PhWRKY23* is not only transcriptionally responsive to herbivore feeding but also functionally contributes to anti-herbivore resistance in *P. hopeiensis*.

These findings are consistent with previous studies showing that WRKY transcription factors play important roles in herbivore-induced defense responses. In *N. attenuata*, WRKY3 and WRKY6 coordinate plant responses to herbivory, and their silencing compromises resistance to herbivore attack [21]. In rice, *OsWRKY70* regulates defense priority and contributes to resistance against chewing herbivores [22]. In the woody plant *B. platyphylla*, *BpWRKY6* enhances insect resistance by regulating JA biosynthesis and terpenoid production [23]. Together with these studies, our results suggest that WRKY transcription factors may play similar roles in plant defense against herbivores in both herbaceous and woody plants, although the specific downstream pathways may differ among species.

Previous studies on WRKY23 have mainly focused on nematode interactions, root development, auxin distribution, flavonol biosynthesis, and PIN polarity regulation [24,25,26]. These studies suggest that WRKY23-like proteins may act at the intersection of developmental regulation and stress responses. Our results extend the known functional scope of WRKY23-like genes by providing evidence that *PhWRKY23* positively contributes to resistance to a chewing herbivore in a woody plant. Therefore, *PhWRKY23* may participate in herbivore-induced transcriptional and defense-related responses in *P. hopeiensis*, although its direct downstream targets and regulatory mechanisms remain to be determined.

### 3.2. Growth-Related and Physiological Traits Associated with *PhWRKY23* Expression

Activation of plant defense is often accompanied by changes in growth and resource allocation. The growth–defense trade-off model proposes that plants must balance limited resources between growth and defense, and strong activation of defense pathways may suppress growth under certain conditions [41]. In this study, stem diameter increments did not differ significantly among WT, overexpression, and RNAi plants, whereas *PhWRKY23*-overexpressing lines generally showed greater internode-length increments than WT and RNAi lines. Together, these results indicate that the enhanced resistance of *PhWRKY23*-overexpressing plants was not accompanied by reductions in the measured growth-related traits under the tested conditions.

This finding is noteworthy because defense activation does not always lead to growth suppression. Previous studies have shown that the relationship between defense activation and plant growth can vary depending on the regulatory gene, physiological state, and environmental context [22,41]. In the present study, the enhanced resistance of *PhWRKY23*-overexpressing plants was accompanied by higher chlorophyll a, chlorophyll b, and carotenoid levels. Chlorophylls are essential for photosynthetic light harvesting, whereas carotenoids contribute to light harvesting, photoprotection, ROS scavenging, and stress adaptation [45,46,47]. The higher chlorophyll and carotenoid levels in *PhWRKY23*-overexpressing plants indicate that *PhWRKY23* expression is associated with photosynthetic pigment accumulation, which may be related to the observed growth and resistance phenotypes.

The physiological data further revealed differences in MDA accumulation among the transgenic and WT plants. RNAi plants accumulated significantly higher MDA levels, indicating greater membrane lipid peroxidation. ROS are important signaling molecules during stress responses, but excessive ROS accumulation can damage membranes, proteins, pigments, and other cellular components [42,43,44]. The higher MDA content observed in RNAi plants suggests that reduced *PhWRKY23* expression is associated with increased membrane lipid peroxidation in *P. hopeiensis*. Together, these findings indicate that suppression of *PhWRKY23* was accompanied by increased membrane lipid peroxidation, whereas *PhWRKY23* overexpression was associated with greater photosynthetic pigment accumulation.

Interestingly, SOD activity, PPO activity, soluble protein content, and soluble sugar content did not differ significantly among genotypes. PPO is frequently associated with anti-herbivore defense because it can oxidize phenolic substrates to quinones, which may reduce food quality or interfere with herbivore digestion [48,49,50]. However, the absence of significant PPO differences indicates that the enhanced resistance of *PhWRKY23*-overexpressing plants may not mainly depend on constitutive PPO activation. Instead, the enhanced resistance of *PhWRKY23*-overexpressing plants was accompanied by increased defense-related hormone accumulation, reduced membrane lipid peroxidation, and higher photosynthetic pigment levels, suggesting the involvement of multiple physiological responses beyond constitutive PPO activity.

### 3.3. *PhWRKY23-Associated* Resistance Is Linked to JA/JA-Ile and SA Accumulation

Phytohormones are central regulators of plant defense against insect herbivores. JA and its bioactive conjugate JA-Ile are particularly important for wound- and chewing herbivore-induced responses [28,29,30,31,32,33,34]. In the present study, JA and JA-Ile levels were highest in *PhWRKY23*-overexpressing plants and lowest in RNAi plants, with WT plants showing intermediate levels. This pattern is consistent with the feeding assay results, in which *PhWRKY23*-overexpressing plants exhibited enhanced resistance, whereas RNAi plants showed increased susceptibility. Therefore, the enhanced resistance associated with *PhWRKY23* overexpression was accompanied by increased JA and JA-Ile accumulation.

SA content also showed a clear genotype-dependent pattern. *PhWRKY23*-overexpressing plants accumulated significantly higher SA levels than WT plants, whereas RNAi plants showed significantly lower SA content. Traditionally, JA signaling is strongly associated with defense against chewing insects and necrotrophic pathogens, whereas SA signaling is more closely associated with resistance to biotrophic pathogens and systemic acquired resistance [34,35,36,37]. However, JA–SA interactions are not simply antagonistic in all cases. Their relationship can be antagonistic, synergistic, or cooperative depending on plant species, stress type, developmental stage, and signal intensity [36,37,38,39]. WRKY transcription factors are important nodes in this hormonal crosstalk; WRKY70, for example, functions as a convergence point for JA- and SA-mediated defense signaling in *Arabidopsis* [57].

The simultaneous increase in JA, JA-Ile, and SA in *PhWRKY23*-overexpressing plants indicates that the enhanced resistance phenotype was associated with the accumulation of both jasmonate- and salicylate-related hormones. Given the established role of JA and JA-Ile in defense against chewing herbivores, their increased accumulation is consistent with the enhanced resistance of *PhWRKY23*-overexpressing plants. The accompanying increase in SA suggests that salicylate-related responses may also be involved in this phenotype. In contrast, ABA content did not differ significantly among WT, overexpression, and RNAi plants, indicating that changes in *PhWRKY23* expression were not associated with detectable differences in ABA accumulation under the tested conditions.

Together, the hormone results demonstrate that the resistance phenotype associated with *PhWRKY23* expression is accompanied by genotype-dependent changes in JA, JA-Ile, and SA accumulation, but not in ABA content. However, whether PhWRKY23 directly regulates genes involved in the biosynthesis or signaling of these hormones remains to be determined. Future dual-luciferase reporter assays, electrophoretic mobility shift assays, and ChIP-qPCR analyses could clarify whether PhWRKY23 directly binds to the promoters of hormone-related genes.

### 3.4. *PhDOX1* Is a Candidate *PhWRKY23-Interacting* Protein in Yeast

Yeast two-hybrid screening identified 40 candidate PhWRKY23-interacting proteins, among which PhDOX1, PhNAC083, and PhSUT1 were considered defense-related candidates. Pairwise yeast two-hybrid validation further showed that PhWRKY23 interacts with PhDOX1 in yeast. PhDOX1 is annotated as an α-dioxygenase, a fatty-acid-metabolism-related enzyme associated with oxylipin biosynthesis. Oxylipins are structurally diverse lipid-derived metabolites that participate in stress signaling, defense responses, and cell death regulation [51,52,53,54,55]. In *Arabidopsis, α-DOX1* has been reported to protect against oxidative stress and cell death [51]. In addition, 9-lipoxygenase- and α-dioxygenase-derived oxylipins contribute to local and systemic defense responses [52]. *α-DIOXYGENASE1* has also been shown to contribute to plant defense against aphids in tomato and *Arabidopsis* [53].

The identification of PhDOX1 as a candidate PhWRKY23-interacting protein provides a potential connection with the physiological and hormonal changes observed in the transgenic plants. PhWRKY23 transgenic plants showed altered MDA accumulation, reflecting differences in membrane lipid peroxidation among genotypes. In addition, *PhWRKY23*-overexpressing plants accumulated higher JA and JA-Ile levels, which are lipid-derived hormones with established roles in defense against chewing herbivores [28,29,30,31,32,33,34]. Because α-dioxygenases also participate in the formation of fatty-acid-derived oxylipins [51,52,53,54,55], the yeast interaction between PhWRKY23 and PhDOX1 identifies PhDOX1 as a candidate for further investigation in relation to these lipid-associated responses. However, the present results do not establish that the changes in MDA, JA, or JA-Ile are mediated by PhDOX1 or that PhWRKY23 and PhDOX1 function in the same defense pathway.

However, the PhWRKY23–PhDOX1 interaction should be interpreted cautiously. Yeast two-hybrid assays demonstrate protein interaction in yeast but do not prove that the same interaction occurs in plant cells. Further experiments, such as bimolecular fluorescence complementation, co-immunoprecipitation, or split-luciferase complementation in plant cells, together with biochemical assays such as pull-down assays, are needed to confirm the PhWRKY23–PhDOX1 interaction and determine its biological relevance. Moreover, because WRKY transcription factors usually regulate downstream genes by binding W-box elements in target promoters [16,17,18,19,20], future promoter-binding and transcriptional activation assays could determine whether *PhWRKY23* also regulates *PhDOX1* or other defense-related genes at the transcriptional level.

Based on these findings, we propose a working model in which *S. litura* feeding induces *PhWRKY23* expression in *P. hopeiensis*. PhWRKY23 predominantly localizes to the nucleus and positively contributes to herbivore resistance. *PhWRKY23*-overexpressing plants exhibited enhanced resistance to *S. litura*, higher JA, JA-Ile, and SA accumulation, higher photosynthetic pigment levels, and lower MDA accumulation than RNAi plants. In contrast, *PhWRKY23* suppression was accompanied by lower defense-related hormone accumulation, higher MDA accumulation, and increased susceptibility to herbivore feeding. The yeast two-hybrid results additionally identify PhDOX1 as a candidate PhWRKY23-interacting protein, although this interaction and its contribution to lipid-derived defense responses require confirmation in planta. Overall, these results suggest that *PhWRKY23* positively contributes to herbivore resistance in *P. hopeiensis* and that this resistance phenotype is associated with changes in defense-related hormone accumulation, membrane lipid peroxidation, and photosynthetic pigment levels. PhDOX1 represents a candidate interactor for future investigation rather than a confirmed component of the PhWRKY23-mediated defense mechanism.

Future studies should identify the direct downstream targets of PhWRKY23 using promoter-binding and transcriptional activation assays and determine whether the observed resistance phenotype depends on JA- and SA-related signaling. In planta validation and functional characterization of the candidate PhWRKY23–PhDOX1 interaction will also be necessary. Furthermore, time-resolved hormone analyses, comprehensive insect life-history assays, and field evaluation will help determine the broader biological and practical significance of *PhWRKY23*-mediated resistance.

## 4. Materials and Methods

### 4.1. Plant Materials and Insect Rearing

Tissue-cultured plantlets of *Populus hopeiensis*, including wild-type (WT), *PhWRKY23*-overexpressing, and RNA interference lines, were cultured on standard subculture medium for approximately 30 d. The plantlets were maintained at 25 ± 2 °C under a 14 h light/10 h dark photoperiod, with a light intensity of 2000–3000 lx. Plantlets with similar growth status and well-developed root systems were transplanted into pots containing a mixture of commercial nutrient soil, vermiculite, and perlite (2:1:1, *v*/*v*/*v*). They were grown in a greenhouse under natural light at approximately 25 °C for 2 months until they reached approximately 15 cm in height. Soil-grown WT and transgenic plants were used for the feeding assays, growth measurements, physiological and biochemical analyses, and phytohormone measurements. Seeds of *Nicotiana benthamiana* used for the subcellular localization assay and the *P. hopeiensis* plant materials were provided by the Forest Genetics and Breeding Laboratory of Inner Mongolia Agricultural University.

Second-instar larvae of *S. litura* (Lepidoptera: Noctuidae) were purchased from Henan Jiyuan Baiyun Industry Co., Ltd., Jiyuan, China. Healthy larvae of similar body size were selected for the feeding assays and starved for 12 h before the experiments to standardize their feeding activity.

### 4.2. Herbivore Feeding Treatments, Transcriptome Analysis, and Candidate Gene Selection

Previously generated transcriptomic data from herbivore-treated *Populus hopeiensis* leaves were used to identify herbivore-responsive genes. The experimental groups included an untreated control (A), a high-intensity *Spodoptera litura* feeding treatment (B), a low-intensity *S. litura* feeding treatment (C), and a mite-feeding treatment (D). For treatments B and C, second-instar *S. litura* larvae were allowed to feed on soil-grown *P. hopeiensis* plants with similar growth status until approximately 1/8 and 1/16 of the leaf area had been consumed within 3 h, respectively. For treatment D, approximately 100 mites were allowed to feed on each plant for 19 h. 3 independent biological replicates were included for each treatment, resulting in a total of 12 RNA-sequencing libraries. The cDNA libraries were sequenced on an Illumina sequencing platform by Metware Biotechnology Co., Ltd. (Wuhan, China). After quality control using fastp v0.23.2, 97.64 Gb of clean data were obtained, with more than 6 Gb generated for each library and Q30 values exceeding 93%. The clean reads were mapped to the *Populus trichocarpa* reference genome obtained from Phytozome (assembly Ptrichocarpa_533_v4.0 with v4.1 gene annotation) using HISAT2 v2.2.1. Gene expression levels were quantified using featureCounts v2.0.3, and differential expression analysis was performed using DESeq2 v1.22.1. Genes with |log_2_(fold change)| ≥ 1 and an FDR < 0.05 were considered differentially expressed. Among the differentially expressed transcription factor genes, *PhWRKY23* showed increased transcript abundance following both levels of *S. litura* feeding and was therefore selected for subsequent functional analysis.

For qRT-PCR validation, second-instar *S. litura* larvae were allowed to feed on soil-grown *P. hopeiensis* plants with similar growth status. Leaves were collected when approximately 1/16, 1/8, or 1/4 of the leaf area had been consumed. Leaves from untreated plants were used as controls. All samples were immediately frozen in liquid nitrogen and stored at −80 °C until RNA extraction. 3 independent biological replicates were used for each treatment.

### 4.3. RNA Extraction, cDNA Synthesis, and qRT-PCR Analysis

Total RNA was extracted from *P. hopeiensis* leaves using the RNAprep Pure Plant Plus Kit (TIANGEN Biotech, Beijing, China) according to the manufacturer’s instructions. RNA concentration and quality were assessed using a spectrophotometer and agarose gel electrophoresis. First-strand cDNA was synthesized using the PrimeScript RT Reagent Kit (TaKaRa, Beijing, China) according to the manufacturer’s instructions.

qRT-PCR was performed using TB Green Premix Ex Taq II (TaKaRa, Beijing, China) on a LightCycler 480 real-time PCR system. Each 20 μL reaction contained 10 μL of TB Green Premix Ex Taq II, 2 μL of cDNA template, 1 μL of forward primer, 1 μL of reverse primer, and 6 μL of ddH_2_O. The amplification program consisted of an initial denaturation at 95 °C for 2 min, followed by 45 cycles of 95 °C for 10 s, 56 °C for 20 s, and 72 °C for 10 s. *ACTIN* was used as the internal reference gene, and relative expression levels were calculated using the 2^−ΔΔCt^ method.

For the herbivory-induced expression analysis, 3 independent biological replicates were analyzed for each treatment, with 3 technical replicates for each biological replicate. For expression analysis of the transgenic lines, leaf samples were collected separately from 3 independently propagated plants of each transgenic line and the WT group. 3 plants were treated as 3 biological replicates (*n* = 3 per line or group), and each biological replicate was analyzed using 3 technical replicates. The primers used in this study are listed in Table S2.

### 4.4. Cloning and Sequence Analysis of PhWRKY23

The full-length coding sequence of *PhWRKY23* was amplified from *P. hopeiensis* leaf cDNA using gene-specific primers (Table S2). The PCR product was purified, cloned, and confirmed by sequencing. The physicochemical properties of the deduced PhWRKY23 protein were analyzed using ExPASy ProtParam online tool (https://web.expasy.org/protparam/, accessed on 15 August 2026).

The amino acid sequences of WRKY23 homologs from different plant species were retrieved from the NCBI database. Multiple sequence alignment was performed using DNAMAN. The conserved WRKY domain, including the WRKYGQK motif and the C_2_H_2_-type zinc-finger motif, was identified based on the sequence alignment. Phylogenetic analysis was performed using MEGA 10. A neighbor-joining phylogenetic tree was constructed with 1000 bootstrap replicates.

### 4.5. Subcellular Localization of *PhWRKY23*

The coding sequence of *PhWRKY23* without the stop codon was inserted into the pBWA(V)HS-ccdB-EGFP vector in-frame with the EGFP coding sequence, generating the *PhWRKY23*-EGFP fusion construct. Expression of the fusion protein was driven by the cauliflower mosaic virus 35S (CaMV 35S) promoter. The empty pBWA(V)HS-ccdB-EGFP vector was used as the control. The recombinant construct and empty control vector were separately introduced into *Agrobacterium tumefaciens* strain GV3101. The vector and GV3101 competent cells were obtained from Wuhan BioRun Biosciences Co., Ltd. (Wuhan, China).

The transformed *A. tumefaciens* cells were resuspended in infiltration buffer containing 10 mM MgCl_2_, and the OD_600_ was adjusted to approximately 0.60. The bacterial suspensions were infiltrated into healthy, fully expanded leaves of one-month-old *Nicotiana benthamiana* plants using a 1 mL needleless syringe. After infiltration, the plants were maintained under low-light conditions for 2 d. EGFP fluorescence signals were subsequently observed using a confocal laser scanning microscope.

### 4.6. Vector Construction and Genetic Transformation of P. hopeiensis

To investigate the function of *PhWRKY23*, overexpression and RNA interference (RNAi) constructs were generated. For overexpression, the full-length coding sequence of *PhWRKY23* was inserted into the pBWA(V)HS-ccdB-EGFP vector under the control of the cauliflower mosaic virus 35S (CaMV 35S) promoter. For RNA interference, a gene-specific fragment of *PhWRKY23* was inserted into the pBWA(V)HS-ccdB-RNAi vector to generate a hairpin RNA construct driven by the CaMV 35S promoter. The recombinant plasmids were verified by PCR and restriction enzyme digestion and subsequently introduced into *Agrobacterium tumefaciens* strain GV3101. The vectors and GV3101 competent cells were obtained from Wuhan BioRun Biosciences Co., Ltd. (Wuhan, China).

Leaf discs of *P. hopeiensis* were transformed using an *Agrobacterium*-mediated transformation method. The transformed *A. tumefaciens* cultures were grown until the OD_600_ reached 0.4–0.6. Young leaves were pre-cultured for 1 d at 28 °C, immersed in the bacterial suspension for 12 min, blotted dry on sterile filter paper, and co-cultivated in the dark at 28 °C for 3 d. After co-cultivation, the explants were transferred to selective differentiation medium for the induction of hygromycin-resistant shoots. Resistant shoots were excised and transferred to selective rooting medium. Rooted plantlets were maintained under sterile tissue-culture conditions and propagated for subsequent analyses.

Putative transgenic plants were initially selected on media containing hygromycin and subsequently verified by genomic PCR using construct-specific primers. The expression level of *PhWRKY23* in the PCR-positive plants was determined by qRT-PCR. Independent overexpression lines showing significantly increased *PhWRKY23* expression and RNAi lines showing significantly reduced expression relative to wild-type plants were selected for subsequent experiments. All media were adjusted to pH 5.9 before sterilization. The selective differentiation and rooting media contained 400 mg L^−1^ Timentin and 3.0 mg L^−1^ hygromycin. Detailed medium compositions are provided in Table S3.

### 4.7. Molecular Identification and Selection of Transgenic Plants

Genomic DNA was extracted from young leaves of putative transgenic plants using a plant genomic DNA extraction kit. Genomic PCR was performed using construct-specific primers (Table S2). Plants producing the expected amplification products of 667 bp and 441 bp were identified as *PhWRKY23*-overexpressing and RNA interference (RNAi) plants, respectively.

The transcript abundance of *PhWRKY23* in the PCR-positive plants was subsequently determined by qRT-PCR, with wild-type (WT) plants used as the control. 3 independent overexpression lines and 6 independent RNAi lines were analyzed. Overexpression lines exhibiting increased *PhWRKY23* transcript abundance and RNAi lines exhibiting reduced transcript abundance relative to WT plants were considered confirmed transgenic lines. Representative independent lines showing pronounced and consistent changes in *PhWRKY23* expression were selected for the feeding assays and subsequent physiological and biochemical analyses.

### 4.8. Choice Feeding Assay

A choice feeding assay was conducted to compare the feeding preference of second-instar *S. litura* larvae among WT, *PhWRKY23*-overexpressing, and RNAi plants. Fully expanded leaves of similar age, size, and developmental status were detached from WT plants and 3 independent overexpression (OE-1, OE-2, and OE-3) and RNAi (RNAi-2, RNAi-3, and RNAi-5) lines. For each biological replicate, one WT leaf, one leaf from an independent overexpression line, and one leaf from an independent RNAi line were placed together in a Petri dish lined with moist filter paper. The leaves were randomly positioned at equal distances from the center of the Petri dish to minimize positional effects.

15 starved second-instar *S. litura* larvae were released into the center of each Petri dish. Leaf damage was photographed at 0, 4, and 8 h after larval release. The consumed leaf area was quantified from digital images using ImageJ software (version 1.54t; National Institutes of Health, Bethesda, MD, USA). 3 independent biological replicates were performed, with each replicate using a different independent overexpression line, a different independent RNAi line, and a separate group of 15 larvae.

### 4.9. No-Choice Feeding Assay

A no-choice feeding assay was conducted to evaluate leaf consumption and larval growth on WT, *PhWRKY23*-overexpressing, and RNAi plants. Fully expanded leaves of similar age, size, and developmental status were collected from WT plants, 3 independent overexpression lines (OE-1, OE-2, and OE-3), and 3 independent RNAi lines (RNAi-2, RNAi-3, and RNAi-5). Leaves from each plant genotype or transgenic line were separately provided to starved second-instar *S. litura* larvae. Each Petri dish was lined with moist filter paper to maintain humidity and minimize water loss from the detached leaves.

For the short-term feeding assay, 5–6 leaves were weighed and placed in a 150 mm Petri dish containing 3 larvae. The leaves provided in each Petri dish were obtained from only 1 genotype or independent transgenic line. Leaf mass loss was determined after 5 h of feeding. For the larval growth assay, 3 larvae in each Petri dish were provided daily with fresh leaves from the corresponding genotype or transgenic line for 6 d. The total mass of the 3 larvae in each dish was recorded before and after the 6 d feeding period, and larval morphology was photographed at the end of the assay. A total of 3 independent biological replicates were performed, with each replicate using a different independent overexpression line, a different independent RNAi line, and an independently prepared WT group.

### 4.10. Measurement of Growth-Related Traits

To evaluate the association between *PhWRKY23* expression and vegetative growth, plant height, basal stem diameter, and internode length were measured in WT plants, 3 independent overexpression lines (OE-1, OE-2, and OE-3), and 3 independent RNAi lines (RNAi-2, RNAi-3, and RNAi-5) with similar initial growth status. The transgenic lines were evaluated separately. For each transgenic line, 3 individual plants were used as biological replicates (*n* = 3 per line), and 3 individual WT plants were used as biological replicates for the WT group (*n* = 3). The same plants were measured at 7-day intervals throughout the experiment. The increment in each trait during each 7-day interval was calculated as the difference between two consecutive measurements.

Plant height was measured from the stem base to the apical growth point using a ruler. Basal stem diameter was measured at a fixed position near the stem base using a vernier caliper. Internode length was measured as the distance between two adjacent nodes.

### 4.11. Determination of Physiological and Biochemical Parameters

Fully expanded functional leaves of similar developmental status were collected from WT plants, 3 independent overexpression lines (OE-1, OE-2, and OE-3), and 3 independent RNAi lines (RNAi-2, RNAi-3, and RNAi-5). The 3 independent overexpression lines and 3 independent RNAi lines were used as biological replicates for the corresponding transgenic groups, and 3 independently grown WT plants were used as biological replicates for the WT group. For each biological replicate, 0.1 g of fresh leaf tissue was used for each measurement. The activities of superoxide dismutase (SOD) and polyphenol oxidase (PPO), as well as the contents of malondialdehyde (MDA), soluble protein, and soluble sugar, were determined using commercial assay kits (Yisejiu Biotechnology Co., Ltd., Lianyungang, China) according to the manufacturer’s instructions.

Absorbance was measured at 560 nm for SOD activity, 525 nm for PPO activity, 532 and 600 nm for MDA content, 540 nm for soluble protein content, and 620 nm for soluble sugar content. Chlorophyll a, chlorophyll b, and carotenoids were extracted with 95% ethanol, and their contents were calculated from the absorbance values measured at 665, 649, and 470 nm according to Minocha et al. [58]. A total of 3 independent biological replicates were analyzed for each group.

### 4.12. Determination of Phytohormone Levels

To determine whether differences in *PhWRKY23* expression were associated with phytohormone accumulation, the levels of jasmonic acid (JA), jasmonoyl-L-isoleucine (JA-Ile), salicylic acid (SA), and abscisic acid (ABA) were measured in WT plants, 3 independent overexpression lines (OE-1, OE-2, and OE-3), and 3 independent RNAi lines (RNAi-2, RNAi-3, and RNAi-5). Fully expanded functional leaves of similar developmental status were collected from plants with similar growth status, immediately frozen in liquid nitrogen, and stored at −80 °C until analysis.

JA, JA-Ile, and SA were quantified using the Plant Jasmonic Acid ELISA Kit (BY-JZF1651), Plant Jasmonoyl-L-isoleucine ELISA Kit, and Plant Salicylic Acid ELISA Kit (BY-JZF1648), respectively, obtained from Nanjing BYabscience Technology Co., Ltd. (Nanjing, China). ABA was quantified using an Abscisic Acid ELISA Quantitative Detection Kit (ml103715; Shanghai Mlbio Biotechnology Co., Ltd., Shanghai, China). The ABA assay was based on an indirect competitive ELISA method.

Fresh leaf tissues were homogenized in phosphate-buffered saline (PBS) and centrifuged, and the resulting supernatants were used for hormone quantification. All assays were performed according to the manufacturers’ instructions. Absorbance was measured at 450 nm using a microplate reader (Thermo Fisher Scientific, Waltham, MA, USA). Standard curves were generated using 4-parameter logistic regression, and hormone levels were calculated from the corresponding standard curves. The 3 independent overexpression lines and 3 independent RNAi lines were used as biological replicates for the corresponding transgenic groups, and 3 independently grown WT plants were used as biological replicates for the WT group.

### 4.13. Yeast Two-Hybrid Autoactivation Assay

The full-length coding sequence of *PhWRKY23* was inserted into the pGBKT7 vector to generate the bait construct pGBKT7-*PhWRKY23*. The recombinant bait construct was verified and introduced into *Saccharomyces cerevisiae* strain AH109. Before library screening, pGBKT7-*PhWRKY23* was tested for transcriptional autoactivation. Yeast transformants were spotted onto SD/−Trp/−Leu, SD/−Trp/−Leu/−His/X-α-gal, and SD/−Trp/−Leu/−His/−Ade/X-α-gal media. The combinations pGBKT7-53 + pGADT7-T and pGBKT7-Lam + pGADT7-T were used as positive and negative controls, respectively. The plates were incubated at 30 °C for 3–4 d.

Because the pGBKT7-*PhWRKY23* bait exhibited weak autoactivation, media containing different concentrations of 3-amino-1,2,4-triazole (3-AT) were tested to determine the concentration required to suppress background growth. Based on yeast growth and color development, 5 mM 3-AT was selected for subsequent library screening and pairwise validation.

### 4.14. Yeast Two-Hybrid Library Screening

The pGBKT7-*PhWRKY23* bait construct was used to screen a cDNA prey library prepared from *P. hopeiensis* leaves subjected to *S. litura* feeding. Yeast cells carrying the bait construct were transformed with the prey library according to the manufacturer’s protocol for the yeast two-hybrid system.

Transformants were selected on SD/−Trp/−Leu/−His/−Ade/X-α-gal medium supplemented with 5 mM 3-AT. Blue colonies showing growth on the selective medium were isolated and subjected to colony PCR. The amplified prey inserts were sequenced, and candidate PhWRKY23-interacting proteins were identified through sequence similarity searches and functional annotation. These proteins were regarded as candidate interactors identified in the yeast system and were subjected to further pairwise validation.

### 4.15. Pairwise Yeast Two-Hybrid Validation

To evaluate the candidate interaction between PhWRKY23 and PhDOX1 in the yeast system, the coding sequence of *PhDOX1* was amplified from *P. hopeiensis* leaf cDNA and inserted into the pGADT7 vector to generate pGADT7-PhDOX1. The recombinant construct was verified by colony PCR.

The pGBKT7-*PhWRKY23* and pGADT7-PhDOX1 constructs were co-transformed into *S. cerevisiae* strain AH109. Transformants were spotted onto SD/−Trp/−Leu, SD/−Trp/−Leu/−His/X-α-gal, and SD/−Trp/−Leu/−His/−Ade/X-α-gal media supplemented with 5 mM 3-AT where appropriate. The pGBKT7-53 + pGADT7-T and pGBKT7-Lam + pGADT7-T combinations were used as positive and negative controls, respectively. Yeast growth and blue coloration were recorded after incubation at 30 °C for 3–5 d. This assay was used to assess the PhWRKY23–PhDOX1 interaction in yeast; confirmation of this interaction in planta requires additional approaches, such as bimolecular fluorescence complementation or co-immunoprecipitation.

### 4.16. Statistical Analysis

All data are presented as means ± standard error (SE). Data were processed using Microsoft Excel 2019 and analyzed using SPSS 25.0. Differences among treatments, groups, or individual transgenic lines, as appropriate, were evaluated using one-way analysis of variance (ANOVA). For growth-related traits measured across different intervals, statistical comparisons were performed separately within each growth interval. When variances were homogeneous, Tukey’s multiple-comparison test was used. When variances were unequal, Welch’s ANOVA was used, followed by an appropriate multiple-comparison procedure where applicable. Differences were considered statistically significant at *p* < 0.05. Different lowercase letters in the figures indicate significant differences among treatments, groups, or individual transgenic lines, as appropriate.

## 5. Conclusions

In this study, *PhWRKY23*, a WRKY transcription factor from *Populus hopeiensis*, was identified as a herbivore-responsive gene induced by *Spodoptera litura* feeding and was predominantly localized in the nucleus. In the choice feeding assay, *PhWRKY23*-overexpressing plants showed less leaf consumption, whereas RNAi plants were consumed more extensively. In the no-choice feeding assay, larvae feeding on the overexpression plants accumulated less mass, whereas those feeding on the RNAi plants accumulated more mass. These findings support a positive contribution of *PhWRKY23* to herbivore resistance in *P. hopeiensis*.

Differences in *PhWRKY23* expression were associated with changes in MDA accumulation, photosynthetic pigment contents, and the levels of JA, JA-Ile, and SA, whereas ABA content did not differ significantly among genotypes. Yeast two-hybrid analysis identified PhDOX1 as a candidate PhWRKY23-interacting protein in yeast, although this interaction and its biological relevance require confirmation in planta. Overall, *PhWRKY23* positively contributes to herbivore resistance in *P. hopeiensis*, but its direct downstream targets and regulatory mechanisms remain to be determined.

## Data Availability

The nucleotide sequence of *PhWRKY23* has been deposited in NCBI GenBank under accession number PV738213. The other data presented in this study are available from the corresponding author upon reasonable request.

## Supplementary Materials

The following supporting information can be downloaded at https://www.mdpi.com/article/10.3390/plants15162483/s1**:** Figure S1: Schematic representation of the predicted domain architecture and candidate NLS regions of PhWRKY23; Figure S2: Autoactivation test of the bait construct pGBKT7-PhWRKY23; Figure S3: Screening of 3-AT concentrations for suppressing PhWRKY23 autoactivation; Table S1: Candidate PhWRKY23-interacting proteins identified by yeast two-hybrid screening; Table S2: Primers used in this study; Table S3: Culture media used for transformation and regeneration of *Populus hopeiensis*; Table S4: Commercial kits and methods used for physiological, biochemical, and phytohormone assays.
