# Supplementary material for: PhWRKY23 Positively Contributes to Herbivore Resistance and Is Associated with Phytohormone and Defense-Related Responses in Populus hopeiensis
Source: Plants (Basel). 2026 Aug 16;15(16):2483. doi: 10.3390/plants15162483 (PMC13516038; doi:10.3390/plants15162483)
Supplement: Supplementary file 1 [file plants-15-02483-s001.zip › Table S1. Candidate PhWRKY23-interacting proteins identified by yeast two-hybrid screening.pdf]

No.	Populus trichocarpa gene ID	Arabidopsis thaliana gene ID	Gene symbol	Putative function
1	Potri.018G130800.v4.1	AT2G25490	EBF1	Protein modification and ubiquitination
2	Potri.017G135600.v4.1	AT4G02890	UBQ14	Ubiquitin
3	Potri.017G036800.v4.1	AT4G05320	UBI10	Ubiquitin
4	Potri.017G030600.v4.1	AT4G24220	VEP1	Wound-induced response
5	Potri.016G127700.v4.1	AT3G51670	PATL6	Carrier protein
6	Potri.014G141500.v4.1	AT1G06460	ACD31.2	Heat-stress-induced response
7	Potri.014G053700.v4.1	AT3G60470	T8B10-130	Transmembrane protein
8	Potri.013G039500.v4.1	AT3G04780	F7018.27	Transcriptional regulation
9	Potri.013G024600.v4.1	AT1G54850	ACD22.3	Alpha-crystallin domain-containing protein
10	Potri.012G069800.v4.1	AT3G47730	ABCA2	Energy transport protein
11	Potri.012G014100.v4.1	AT4G27890	BOB2	Cotyledon primordium growth
12	Potri.012G002600.v4.1	AT5G53120	SPMS	Spermine biosynthesis
13	Potri.011G134200.v4.1	AT4G02890	UBQ14	Ubiquitin
14	Potri.010G013400.v4.1	AT3G16640	TCTP1	Growth and development
15	Potri.008G106400.v4.1	AT3G01420	DOX1	Antifungal and antibacterial stress responses
16	Potri.007G123300.v4.1	AT4G02890	UBQ14	Ubiquitin
17	Potri.007G115900.v4.1	AT5G64370	PYD3	Pyrimidine degradation
18	Potri.006G254100.v4.1	AT2G25490	FBL6	Ubiquitin-related process
19	Potri.006G052800.v4.1	AT5G20250	RFS6	Sugar biosynthesis
20	Potri.006G041900.v4.1	AT3G57090	FIS1A	Organelle division
21	Potri.006G031700.v4.1	AT2G40840	DPE2	Cytosolic maltose metabolism
22	Potri.005G257200.v4.1	AT1G76500	AHL29	Hypocotyl and leaf growth
23	Potri.005G241700.v4.1	AT1G20080	SYT2	Protein transport from the cytoplasm to the extracellular matrix
24	Potri.005G223600.v4.1	AT3G54700	PHT1-7	Inorganic phosphate transporter
25	Potri.005G164500.v4.1	AT1G22300	GRF14	Transcriptional regulation
26	Potri.005G021400.v4.1	AT4G11420	TIF3A1	Eukaryotic translation
27	Potri.004G115900.v4.1	AT3G01090	KIN10	Proteasome-related ubiquitination and cell proliferation
28	Potri.003G202500.v4.1	AT4G14270	CID2	Poly(A)-binding protein-interacting protein
29	Potri.003G114900.v4.1	AT4G12230	TIF3A1	Translational initiation / protein synthesis
30	Potri.003G097400.v4.1	AT1G32160	DUF760-1	Photosynthesis
31	Potri.003G054900.v4.1	AT4G19640	RABF2B	Endocytosis
32	Potri.002G257500.v4.1	AT3G04400	RPL23A	Protein synthesis
33	Potri.002G221400.v4.1	AT2G05070	LHCB2.2	Photosynthesis
34	Potri.002G097500.v4.1	AT1G22300	GRF10	Transcriptional regulation
35	Potri.002G040600.v4.1	AT1G06150	EMB1444	Root development
36	Potri.002G003700.v4.1	AT1G76510	ARID5	RNA transcription
37	Potri.001G418500.v4.1	AT4G05050	UBQ11	Ubiquitin

38	Potri.001G325100.v4.1	AT5G13180	NAC083	Viral infection-related DNA replication
39	Potri.001G313300.v4.1	AT5G63020	SUT1	Plant immune response
40	Potri.001G263000.v4.1	AT4G05320	UBQ10	Ubiquitin

Table S1. Candidate PhWRKY23-interacting proteins identified by yeast two-hybrid screening. Candidate proteins were identified by yeast two-hybrid screening using PhWRKY23 as the bait protein. Functional annotations were inferred from homologous genes in *Arabidopsis thaliana* and available gene annotation information.
