# Supplementary material for: PhWRKY23 Positively Contributes to Herbivore Resistance and Is Associated with Phytohormone and Defense-Related Responses in Populus hopeiensis
Source: Plants (Basel). 2026 Aug 16;15(16):2483. doi: 10.3390/plants15162483 (PMC13516038; doi:10.3390/plants15162483)
Supplement: Supplementary file 1 [file plants-15-02483-s001.zip › Table S2. Primers used in this study.pdf]

| Purpose | Primer name | Primer sequence (5′–3′) |
|---|---|---|
| Cloning of *PhWRKY23* | WRKY23-F | ATGGAGAGGAGAGAAGTTGTGAAGGTTG |
| | WRKY23-R | CTCTTCCTTTAGCATATGGGAGGGG |
| qRT-PCR of *PhWRKY23* | WRKY23-RT-F | TCAGTGATCCAAGCGTAGTTG |
| | WRKY23-RT-R | ATCAGATGCAGGTCCAGTAAAG |
| qRT-PCR reference gene | ACTIN-F | AGGCAGGTTTCGCAGGAGATGA |
| | ACTIN-R | TCCATCACCAGAATCCAGCACA |
| Colony PCR identification of overexpression vector | WRKY23-G-F | TTCATTTGGAGAGAACACGGGGGAC |
| | WRKY23-G-R | GACCTTCATAAGTGGTCACAAC |
| Colony PCR identification of RNAi vector | WRKY23-GR-F | TTCATTTGGAGAGAACACGGGGGAC |
| | WRKY23-GR-R | GGGCTCTGTAACTATC |
| Construction of RNAi sense fragment | RNAi-F | CAGTGGTCTCACAACCCGGCATTTAGTACTACTAAT GATCATGTTCAAAC |
| | RNAi-R | CGATGGTCTCACAGGGCGACTCCGTAACTACTTGAA CATTTGG |
| Construction of loop fragment | Loop-F | CGATGGTCTCACCTGCAGGTCTAGTTTTTCTCCTTCA TTTTC |
| | Loop-R | CGATGGTCTCAGCCCGGGCTCTGTAACTATCATC |
| Construction of RNAi antisense fragment | RNAi2-F | CAGTGGTCTCAGGGCGCGACTACGTAACTACTTGAA CATTTGG |
| | RNAi2-R | CAGTGGTCTCATACACCGGCATTTAGTACTACTAAT GATCATGTTCAAAC |
| Amplification of *PhWRKY23* for bait vector construction | WRKY23-Y-F | TGATCTCAGAGGAGGACCTGATGGAGAGGAGAGAA GTTGTGAAGGTTG |
| | WRKY23-Y-R | GCCCCAAGGGGTTATGCTAGTTACTCTTCCTTTAGCA TATGGGAGGGG |
| Colony PCR identification of bait vector | WRKY23-YJ-F | GACAGTTGACTGTATCGCC |
| | WRKY23-YJ-R | CCTTCATAAGTGGTCACAACTAC |
| Amplification of *PhDOX1* for prey vector construction | DOX1-L-F | ATATGGCCATGGAGGCCAGTGAATTC |
| | DOX1-L-R | GAGAAGTTCAACAGTCCAATC |

**Table S2. Primers used in this study.**All primer sequences are listed in the 5′–3′ direction. *ACTIN* was used as the internal reference gene for qRT-PCR. Gene names are italicized, whereas primer names are not italicized.
