# Supplementary material for: PhWRKY23 Positively Contributes to Herbivore Resistance and Is Associated with Phytohormone and Defense-Related Responses in Populus hopeiensis
Source: Plants (Basel). 2026 Aug 16;15(16):2483. doi: 10.3390/plants15162483 (PMC13516038; doi:10.3390/plants15162483)
Supplement: Supplementary file 1 [file plants-15-02483-s001.zip › Table S3. Culture media used for transformation and regeneration of Populus hopeiensis.pdf]

Medium	Basal medium	Plant growth regulators	Antibiotics	Main use
WT rooting medium	1/2 MS	0.30 mg L^{-1} IBA + 0.05 mg L^{-1} NAA	-	Rooting and propagation of wild-type plantlets
Co-cultivation medium	MS	0.30 mg L^{-1} TDZ + 0.40 mg L^{-1} IBA	-	Co-cultivation of leaf explants with *Agrobacterium tumefaciens*
Selective shoot induction medium	MS	0.30 mg L^{-1} TDZ + 0.40 mg L^{-1} IBA	400 mg L^{-1} Timentin + 3.00 mg L^{-1} hygromycin	Selection and induction of resistant shoots
Selective rooting medium	1/2 MS	0.30 mg L^{-1} IBA + 0.05 mg L^{-1} NAA	400 mg L^{-1} Timentin + 3.00 mg L^{-1} hygromycin	Rooting of resistant transgenic shoots

Table S3. Culture media used for transformation and regeneration of *Populus hopeiensis*. MS, Murashige and Skoog medium; IBA, indole-3-butyric acid; NAA, 1-naphthaleneacetic acid; TDZ, thidiazuron. The pH of all media was adjusted to approximately 5.9 before sterilization. Timentin was used to suppress *Agrobacterium* growth, and hygromycin was used for selection of transgenic plantlets.
