# Supplementary material for: PhWRKY23 Positively Contributes to Herbivore Resistance and Is Associated with Phytohormone and Defense-Related Responses in Populus hopeiensis
Source: Plants (Basel). 2026 Aug 16;15(16):2483. doi: 10.3390/plants15162483 (PMC13516038; doi:10.3390/plants15162483)
Supplement: Supplementary file 1 [file plants-15-02483-s001.zip › Table S4. Commercial kits and methods used for physiological, biochemical, and phytohormone assays.pdf]

Assay item	Kit or method	Supplier	Detection principle / method	Detection wavelength
Polyphenol oxidase (PPO) activity	PPO assay kit	Yisejiu Biotechnology, China	Colorimetric assay	525 nm
Superoxide dismutase (SOD) activity	SOD assay kit	Yisejiu Biotechnology, China	Nitroblue tetrazolium method	560 nm
Malondialdehyde (MDA) content	MDA assay kit	Yisejiu Biotechnology, China	Colorimetric assay	532 and 600 nm
Soluble protein content	Soluble protein assay kit	Yisejiu Biotechnology, China	Biuret method	540 nm
Soluble sugar content	Plant soluble sugar assay kit	Yisejiu Biotechnology, China	Colorimetric assay	620 nm
Chlorophyll a content	95% ethanol extraction	-	Spectrophotometric assay	665 and 649 nm
Chlorophyll b content	95% ethanol extraction	-	Spectrophotometric assay	665 and 649 nm
Carotenoid content	95% ethanol extraction	-	Spectrophotometric assay	470, 665, and 649 nm
Jasmonic acid (JA) concentration	Plant jasmonic acid ELISA kit	Nanjing BYabscience Nanjing BYabscience Technology Co., Ltd., China	Competitive ELISA	450 nm
Jasmonoyl-L-isoleucine (JA-Ile) concentration	Plant JA-Ile ELISA kit	Commercial kit	Competitive ELISA	450 nm
Salicylic acid (SA) content	Plant salicylic acid ELISA kit	Nanjing BYabscience Technology Co., Ltd., China	Competitive ELISA	450 nm
Abscisic acid (ABA) content	ABA ELISA quantitative detection kit	Shanghai Mlbio Biotechnology Co., Ltd., China	Indirect competitive ELISA	450 nm

Table S4. Commercial kits and methods used for physiological, biochemical, and phytohormone assays. All physiological and biochemical assays were performed using fresh leaf samples according to the manufacturers' instructions. Chlorophyll a, chlorophyll b, and carotenoid contents were determined by 95% ethanol extraction rather than commercial kits. JA, JA-Ile, SA, and ABA were quantified using ELISA-based methods, and standard curves were generated for hormone calculation. "Not provided" indicates that the catalog number was not available in the provided kit manual.
